# How solvent determines the molecular reactive conformation and the selectivity: Solvation spheres and energy

**DOI:** 10.3389/fchem.2022.1012769

**Published:** 2022-09-29

**Authors:** Joseelyne Hernández-Lima, Karla Ramírez-Gualito, Beatriz Quiroz-García, Ana Luisa Silva-Portillo, Ernesto Carrillo-Nava, Fernando Cortés-Guzmán

**Affiliations:** ^1^ Instituto de Química, Unversidad Nacional Autónoma de México, Coyoacan, Mexico; ^2^ Centro de Nanociencias y Micro y Nanotecnologías, Instituto Politécnico Nacional, Ciudad de Mexico, México; ^3^ Facultad de Química, Unversidad Nacional Autónoma de México, Mexico City, Mexico

**Keywords:** solvent effect, solvent spheres, explicit solvent, reactive conformation, solute-solvent interaction

## Abstract

In solution, the solvent determines the molecular conformation and the chemical reaction viability and selectivity. When solvent-solute and solvent-solvent interactions present similar strengths, explicit salvation is the best way to describe a system. The problem to solve is how big the explicit shell should be. In this paper, we want to answer one of the fundamental questions in the implementation of explicit solvation, exactly how many solvent molecules should be added and where they should be placed. Here we determine the first solvent sphere around a molecule and describe how it controls the conformation and selectivity of a selected reaction. NMR experiments were carried out to identify the number of solvent molecules around the solute that constitutes the first solvent sphere, and the interaction between this solvent sphere and the solute was detected using DFT and QTAIM calculations. A new approach to the solvation energy is presented. Finally, we established the role of solvent molecules in the conformation of the solute and in the transition states that produce the two possible products of the reaction.

## 1 Introduction

Several factors determine the path a chemical reaction follows; some depend on the structural and electronic features of the reactants, but the reaction environment controls others. In solution, the solvent determines the molecular conformation and, therefore, the reaction viability and selectivity. There are several examples of the influence of the solvent on the conformation and the reactivity of organic molecules. (Reichardt and Welton, 2011) In this work, we present an NMR approach to determine the first and the external solvation spheres of a molecule and a theoretical approach to calculate the explicit solvation energy. We also present the analysis of the specific interactions between the reactant and its solvent sphere, which controls the conformation and selectivity of a reaction.

Usually, the solvent effect is modeled by continuous models, where the system is composed of a single solute molecule immersed in an infinite solvent reservoir. The solvent is considered isotropic, and the solute-solvent interactions are limited to those of electrostatic origin, and usually, no dynamic effects are considered. (Tomasi et al., 2005) Implicit solvation uses a molecular cavity following the geometry of the system and a surface charge distribution to represent the polarization of the environment. (Floris et al., 1991) This approach ignores the short-range effects of hydrogen bonding and other weak interactions. In the case of nonpolar solvents, with low dielectric constant and small dipole moment, like hexane, cyclohexane, benzene, toluene, or chloroform, the implicit models do not provide higher accuracy than gas-phase calculations. The main shortcoming of continuum models is their failure to account for chemical interactions between solute and solvent molecules in the first solvation shell, especially for solutes with hydrophobic groups, (da Silva et al., 2009) where the solvent-solvent and solvent-solute interactions have similar strength. In some cases, despite using an accurate implicit solvent model, specific solute-solvent interactions play an important role in the solution, where the addition of explicit solvent molecules (micro-solvation) significantly improves the accuracy of the properties prediction. This is the case of pKa, where the presence of the hydrogen bonding of a single water molecule is necessary to obtain a correlation with the experiments. (Kelly et al., 2006) Another example is the calculation of the electron paramagnetic resonance spectrum, where the addition of several solvent molecules helps to reduce the difference with the experimental data. (Grande-Aztatzi et al., 2013) To approximate the bulk water’s role in the solution-phase proton-transfer processes, Bayse and others have included clusters of explicit water molecules in the gas phase to provide an indirect pathway for the proton exchange assisted by the solvent. (Bayse, 2007; Bayse and Antony, 2009; Bayse, 2010; Bayse, 2011).

Chemical reactions may involve species more complex than just molecules of reagents. The chemical reactions occur in non-ideal solutions and not between the isolated molecules of reagents. The presence of various supramolecular species, including solution domains, solute clusters, solute aggregates, and supramers, along with non-aggregated solute molecules should be considered (Kononov, 2015). The solute aggregates may differ dramatically in their chemical properties from those of isolated molecules, the latter being commonly considered by traditional organic chemistry textbooks as the genuine chemical properties of the corresponding compound. The trade-off between the inaccuracies of implicit solvent models and the computational cost of explicit models is an important issue that produces several methods which provide a reasonable accuracy at an appropriate cost. Combined implicit–explicit hybrid models present a central part of the system that contains explicit solute and a few explicit solvent molecules, and the rest of the system is treated as a dielectric continuum, also called the cluster-continuum model (Sunoj and Anand, 2012; Skyner et al., 2015). On the other hand, a solute can be embedded in a solvent box and optimized with hybrid methods such as ONIOM (Chung et al., 2015) and QM/MM (Bulo et al., 2009), where some solvent molecules are included in the higher level. In these cases, the question remains valid, how many solvent molecules include within the solute level. For example, Boereboom et al. employed two spherical QM regions encompassing the two reactive moieties in the reactant, while the micro-solvation model confined five QM water molecules near a reactive site at all times, where the dual-sphere explicit solvation allows a superior description of solvent rearrangement along the entire reaction path. (Boereboom et al., 2018) Recently Hasen et al. investigated how the solvation influences the competition between the S_N_2 and E2 pathways of the model F^−^ + C_2_H_5_Cl reaction. In this case, the system was solvated in a stepwise manner by going from the gas phase, then *via* micro-solvation of one to three explicit solvent molecules, then last to bulk solvation. (Hansen et al., 2022) Karmeling et al. showed that when the solvent conformations are chosen at random mixed explicit/implicit solvation models, the phosphate hydrolysis reaction barriers are much larger than the experimental one. To obtain the acceptable values, it would be necessary to sample over many solvent configurations (Kamerlin et al., 2009).

One of the fundamental questions in implementing explicit solvation is exactly how many solvent molecules should be added and where they should be placed (Tantillo, 2018). Another important question is to determine the role of the explicit solvent molecules with active participation during the reaction or just as a spectator. In this work, we present the experimental determination of the first (FSS) and external (ESS) solvent spheres around a molecule, a theoretical approach to the explicit solvation energy and the analysis of the specific interaction within it, the role of the subsequent solvent spheres, and the description of how FSS controls the conformation and selectivity of a reaction. *FSS can be defined as the set of solvent molecules that are the first neighbors of the solute. ESS can be defined by the group containing all solvent molecules which are bonded to the FSS fixing their positions around the solute* (Schienbein et al., 2017).

To explore these ideas, we selected a known chemical reaction whose selectivity depends on solvents with low dielectric constant and small dipole moment, where the implicit models do not account for the difference in the product ratios. This is the case of the epoxy-nitrile cyclization, a reaction developed by Gilbert Stork, which gives one of two possible rings after an intramolecular nucleophilic attack by the *α*-nitrile-carbanion to an oxirane ring (Stork et al., 1974). Baldwin’s rules for opening this kind of three-membered ring to form cyclic structures seem to lie between those for tetrahedral and trigonal systems, generally preferring Exo modes. (Baldwin, 1976) Stork found that the *δ*-epoxy-nitrile cyclization prefers to give cyclobutanes to cyclopentanes, as expected by Baldwin’s rules, leading to a general and non-photochemical synthetic method of substituted cyclobutanes as the (±)-Grandisol (Stork and Cohen, 1974). Stork considered that the oxirane ring imposes a rigidity on the system, which makes it difficult to achieve the proper collinear arrangement for an SN_2_ attack to give a five-membered ring, whereas the transition state for the formation of a four-membered ring seems to allow easy attainment of collinearity. However, there are examples where one can observe the formation of a cyclopentane rather than a cyclobutane ring, as in the cases of the total syntheses of (±)-*α*-Cuparenone and (±)-Epilaurene (Avila-Zárraga and Maldonado, 2000). Luján-Montelongo et al. found that the cyclizations of *δ*-epoxy-nitrile are also controlled through the choice of cation within the alkyl metal base and the solvent (Fleming et al., 2008; Lujan-Montelongo et al., 2009). For example, using lithium hexamethyldisilylamide (LiHMDS) as the base, the reaction prefers a four-membered ring with benzene as the solvent, whereas toluene changes the preference to a five-membered ring. This work shows how the specific solute-solvent interactions determine the molecular conformation of the solute and then the selectivity of a reaction, in this case, the cyclization of *δ*-epoxy nitriles.

## 2 Methodology

### 2.1 Experimental methods

#### 2.1.1 Materials

Most chemicals were obtained from commercial sources and used as received. Solvents such as ethyl acetate, hexane, acetone, dichloromethane, were distilled before being used. Benzene, toluene, and tetrahydrofuran (THF) were used freshly distilled from metallic sodium. Deuterated solvents were obtained from Cambridge Isotope Laboratories Inc. Lithium hexamethyldisilylamide (LiHMDS) was used as a standard commercial solution in toluene from Sigma-Aldrich. Laboratories Flash chromatography was carried out on hand-packed silica gel 60 (230–400 mesh) columns. Merck TLC silica gel 60 F254 aluminum plates were used for thin-layer chromatography. NMR spectra were acquired using a Bruker Avance III 750 MHz (17.6 Tesla) equipped with a TCI triple resonance cryoprobe and in a Varian-Inova 500 MHz. Chemical shifts are reported in ppm (*δ*) and referenced to TMS (*δ*H = 0 ppm). The spectral data required for the study comprise 1D (^1^H–NMR, ^13^C–NMR and titrations) and 2D (COSY, HSQC, HMBC, NOESY). Calorimetric data were obtained using a MicroCal VP-ITC.

#### 2.1.2 Synthesis

The synthesis of the 2-phenyl-6,6-dimethyl-5-epoxynitrile 1) was performed by the alkylation-oxidation procedure reported by Lujan-Montelongo *et al.* report (Lujan-Montelongo et al., 2009). The alkylation reaction of the phenylacetonitrile with 5-iodo-2-methyl-pent-2-ene was performed in THF at -78°C, using lithium diisopropylamide (LDA) as base to give 2-phenyl-6-methylhept-5-enonitrile. Epoxidation of the alkylated nitrile was then done with meta-chloroperoxybenzoic acid (MCPBA) as oxidant to obtain 4-(3,3-dimethyloxiran-2-yl)-2-phenylbutanenitrile (1). The intermolecular cyclization reactions were performed in benzene or toluene using (LiHMDS) as base.

#### 2.1.3 Characterization

The characterization of racemic 2-phenyl-6-methylhept-5-enonitrile, 4-(3,3-dimethyloxiran -2-yl)-2-phenylbutanenitrile (1), 2-(2-hydroxypropan-2-yl) 1-phenylcyclobutane-1- carbonitrile, (4MR) and 3-hydroxy-2,2- dimethyl-1-phenylcyclopentane-1-carbonitrile, (5MR) were performed by NMR techniques and compared with a previous report of these compounds (Lujan-Montelongo et al., 2009).

*2-phenyl-6-methylhept-5-enonitrile*. ^1^H–NMR (500 MHz, CDCl_3_): *δ* ppm 7.67–7.16 (m, 5H), 5.07 (tdt, J = 5.7, 2.8, 1.4 Hz, 1H), 3.78 (dd, J = 8.8, 6.2 Hz, 1H), 2.28–2.08 (m, 2H), 2.08–1.78 (m, 2H), 1.71 (q, J = 1.3 Hz, 3H), 1.61 (d, J = 1.3 Hz, 3H). ^13^C–NMR (125 MHz, CDCl_3_): *δ* ppm 134.96, 133.80, 129.01(2C), 128.22, 127.31 (2C), 122.01, 119.98, 36.98, 36.01, 25.6, 25.78, 16.99.

*4-(3,3-dimethyloxiran-2-yl)-2-phenylbutanenitrile* (1). ^1^H–NMR (750 MHz, CDCl_3_): *δ* ppm 7.33–7.24 (m, 10H), 3.86–3.80 (m, 2H), 2.66 (ddd, J = 14.7, 7.8, 4.7 Hz, 2H), 2.15–1.90 (m, 4H), 1.83–1.73 (m, 1H), 1.66 (dddd, J = 14.2, 9.5, 6.1, 4.8 Hz, 1H), 1.62–1.54 (m, 1H), 1.54–1.45 (m, 1H), 1.23 (s, 6H), 1.20 (s, 3H), 1.15 (s, 3H). ^13^C–NMR (187.5 MHz, CDCl_3_): *δ* ppm 137.00, 136.68, 130.52 (2C), 130.49 (2C), 129.58, 129.55, 128.76 (2C), 128.53(2C), 121.96, 121.84, 64.71, 64.37, 59.78, 59.67, 38.66, 38.05, 34.62, 34.14, 27.94, 27.23, 26.08, 26.06, 20.09, 20.07.

*2-(2-hydroxypropan-2-yl) 1-phenylcyclobutane-1- carbonitrile*, (4MR). ^1^H–NMR (750 MHz, CDCl_3_): *δ* ppm 7.61 (dd, J = 7.6, 1.7 Hz, 2H), 7.34 (dt, J = 53.1, 7.6 Hz, 3H), 3.06 (dd, J = 11.0, 8.9 Hz, 1H), 2.73 (ddd, J = 12.4, 10.7, 8.9 Hz, 1H), 2.64 (ddd, J = 12.1, 9.1, 2.6 Hz, 1H), 2.47 (qd, J = 11.0, 9.1 Hz, 1H), 2.12 (dtd, J = 11.5, 8.9, 2.6 Hz, 1H), 1.71–1.41 (m, 1H), 1.04 (s, 3H), 0.86 (s, 3H). ^13^C–NMR (187.5 MHz, CDCl_3_): *δ* ppm 133.58, 128.07 (2C), 127.52, 126.98 (2C), 123.30, 70.55, 54.66, 41.57, 29.02, 26.24, 26.04, 19.21.

*3-hydroxy-2,2-dimethyl-1-phenylcyclopentane-1-carbonitrile*, (5MR). ^1^H–NMR (750 MHz, CDCl_3_): *δ* ppm 7.49 (td, J = 7.2, 1.3 Hz, 1H), 7.43–7.36 (m, 4H), 4.30 (t, J = 8.4 Hz, 1H), 2.67 (ddd, J = 13.9, 11.9, 6.5 Hz, 1H), 2.38 (dtd, J = 13.6, 9.3, 6.4 Hz, 1H), 2.21 (ddd, J = 13.9, 9.6, 4.2 Hz, 1H), 1.73–1.68 (m, 1H), 1.13 (s, 4H), 0.51 (s, 3H). ^13^C–NMR (187.5 MHz, CDCl_3_): *δ* ppm 134.99, 128.30(2C), 128.28, 127.68(2C), 123.93, 79.56, 54.22, 49.21, 29.71, 28.98, 21.22, 14.61.

The assignment of the 1, 4MR, and 5MR NMR signals using the 2D techniques are detailed in the supplementary information.

#### 2.1.4 Determination of the solvation spheres

##### 2.1.4.1 Anisotropic titration

The ^1^H–NMR titration curve was carried out in CDCl_3_ with benzene-*d*_6_ and toluene-*d*_8_ as titration solvents. 1 (15 mg, 0.06 mmol) was dissolved in CDCl_3_ (0.75 ml), then additions of aromatic deuterated solvent (benzene-*d*_6_ or toluene-*d*_8_, 0 to 7 equivalent) were done before each ^1^H–NMR measure. Chemical shifts were used to establish the first solvent sphere. The data is available in supporting information. The number of molecules within the first solvation sphere was achieved by the second derivative of the chemical shifts with respect to the equivalents of an added aromatic solvent. The slope of a titration curve reaches its maximum value at the inflection point. The first derivative of a titration curve shows a separate peak for each end point. The first derivative is approximated as Δ*δ*/Δ*V*, where Δ*δ* is the change in the chemical shift between successive additions of titrant solvent. The second derivative of a titration curve, Δ^2^ *δ*/Δ*V* ^2^, is more useful than the first derivative since the endpoint is indicated by its intersection with the volume axis. (Harvey, 1999; Denney et al., 2000). The same procedure was carried out for 4MR and 5MR.

##### 2.1.4.2 Calorimetric measurements

Using the technique of Isothermal Titration Calorimetry (ITC) it is possible to determine the energy evolved (Δ_*trans*_ *H*) when transferring the solute dissolved in chloroform to benzene or toluene. Since experimentally, the transfer of the solute also involves the mixing process of chloroform with benzene or toluene (Δ_*obs*_ *H*), we performed the titration of pure chloroform into benzene and toluene in separate experiments from where the energy of mixing of the solvents (Δ_*solv*_ *H*) is determined and can be subtracted from the titration of toluene or benzene with the solute dissolved in chloroform to calculate the Δ_*trans*_ *H*:

$$\Delta_{\mathrm{trans}}H=\Delta_{\mathrm{obs}}H-\Delta_{\mathrm{solv}}H$$ (1)

Experimentally the cell of the calorimeter is filled either with benzene or toluene while the syringe of the calorimeter is filled with either pure chloroform (for the experiments from which Δ_*solv*_ *H* will be determined) or with a 32 mM solution of the solute dissolved in chloroform (for the experiments from which Δ_*trans*_ *H* will the calculated). Additions of 1 *μ*L of the solution placed in the syringe are introduced into the cell. The additions produce an evolution of heat and therefore a change in temperature in the cell which the calorimeter compensates with a change in power to keep the temperature constant. The power signals (peaks) are integrated to obtain the corresponding heats associated with each of the solution additions placed in the syringe into the calorimeter cell with the Origin 7.0 software macros supplied with the calorimeter. All the experiments were done at a temperature of 298.15 K.

#### 2.1.5 Computational methods

All structures were optimized at M062X (Zhao and Truhlar, 2008)/6-31(d,p) level with Gaussian 09 (Frisch et al., 2009). To obtain NMR spectroscopy data, the GIAO method (Ditchfield, 1974) was used at the same level. Three molecular environments utilized gas phase, implicit and hybrid solvation, to understand the role of specific solute-solvent interactions. Conductor-like polarisable continuum model, CPCM, was the implicit method chosen as a continuous medium for benzene and toluene solvent (Cossi et al., 2003). Vibrational frequency calculations were performed to characterize the nature of the stationary points. As reported, the initial geometries were established using experimental observation, where the lithium cations link oxygen and nitrogen atoms of **1**. For mixed solvation (explicit-implicit), we considered conditions to obtain a model that adequately described the specific solute-solvent interactions and whose calculation level was not computationally expensive: 1) The reactant, products, and transition states were first obtained in the gas phase, and then they were surrounded by solvent molecules. 2) The data from the anisotropic titrations were used to locate the position of the first solvation sphere. 3) Due to the diffusion of the solvent molecules in the medium, it was decided to involve a greater number of solvent molecules until the NMR spectra were reproduced. The capability of the systems to reproduce the experimental chemical shifts was monitored by comparing the experimental with the theoretical data of 1, 4MR and 5MR. To increase the solvent molecules, a cluster of 30 benzene molecules was used as reported by Llanio-Trujillo et al. (2011) 4) We included two lithium cations linked to the nitrogen and oxygen atoms due to the experimental conditions including two equivalents of LDA base. 5) Finally, the set of Kohn–Sham orbitals obtained for each system in the different solvation situations was used to calculate the atomic properties using the AIMAll program package to analyze the solute-solvent interactions. (Keith, 2019).

#### 2.1.6 Explicit solvation energy

The solvation energy reflects the environmental change around a solute from vacuum to a solvent, accounting for the cavity formation, the dipole orientation, electrostatic and specific interactions. (Pure and Chemistry, 2009) In this work, we propose a new theoretical approach to the solvation energy (*E*_*solv*_) based on the knowledge of the solvation spheres. The calculation of *E*_*solv*_ two steps: 1) determination of the energy change associated with the formation of the solvent cavity from an optimized solvent cluster, and 2) the energy change of the solute from the gas phase to the explicit solvation, as shown in Eq. (2). In this approach, all the factors involved in the solvation are obtained from the explicit solute-solvent interactions and the cavity is not just an electrostatic space but a real hole within a solvent cluster.

$$\begin{array}{ll} & \;\;\;\;\;\;\;E_{\mathrm{solv}}=\Delta E_{\mathrm{cavity}}+\Delta E_{\mathrm{solute}}\\ & \Delta E_{\mathrm{cavity}}=E_{\mathrm{solvent-cavity}}-E_{\mathrm{optimized-solvent}}\\ & \Delta E_{\mathrm{solute}}=E_{\mathrm{solvated-solute}}-E_{\mathrm{gas-phase-solute}} \end{array}$$ (2)

## 3 Results and discussion

### 3.1 First solvation sphere determination

**FIGURE 1:**
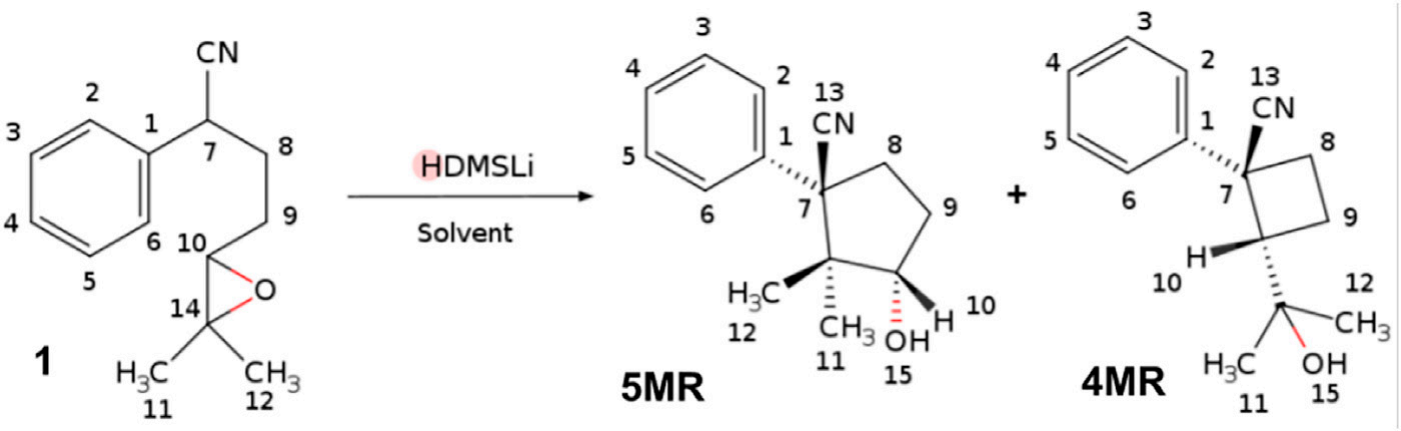
Cyclization of the *δ*-epoxy nitriles 1 to give 4MR and 5MR. Atomic labels are shown for the three structures.

**FIGURE 2:**
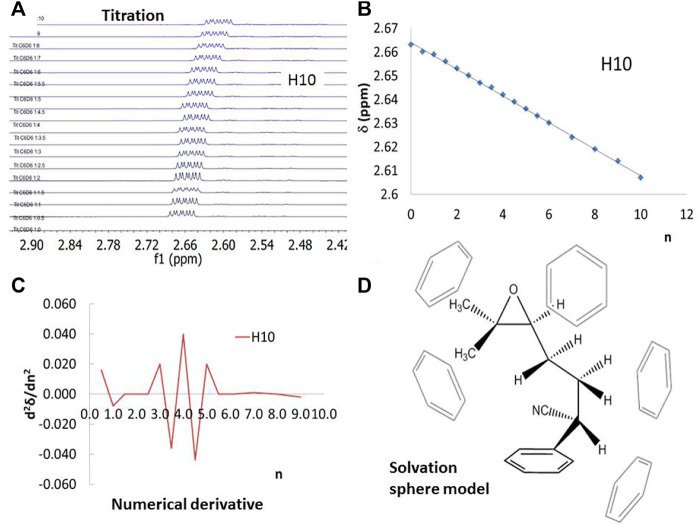
^1^H–NMR of 1 using benzene-*d*_6_ as titration solvent to determine the number of solvent molecules that provoke chemical shift changes. **(A)** and **(B)** H_10_ Chemical shift evolution during the anisotropy titration, **(C)** the second derivative of the chemical shift with respect to the solvent equivalents, **(D)** solvent sphere model extracted from the NMR titration.

To establish the composition of the first solvation sphere of each structure in the aromatic solvents, the ^1^H–NMR signals of 1, 4MR and 5MR in CDCl_3_, benzene, and toluene were compared to find the useful chemical shifts to understand the solvation changes (see S.I. for details). The chemical shift of protons H_7_, H_8_, H_9_, and H_10_ gives more positive values than the other protons of the three structures (see Table 1 and Figure 1 for labels). Then we performed NMR anisotropy titrations using benzene-*d*_6_ or toluene-*d*_8_, to determine the number of solvent molecules that provoke chemical shift changes, which constitute the first solvation spheres. The NMR titrations have been used for monitoring intermolecular aromatic solvent–sugar interactions. (Vandenbussche et al., 2008) A sample of an anisotropy titrations is presented in Figure 2. The number of solvent molecules was achieved by the second derivative of the chemical shifts concerning the equivalents of an added aromatic solvent (see S.I. for details). In this way, it was found that 1 is surrounded by five benzene molecules or four toluene molecules in their first solvation spheres (Figure 2D). The NMR information was useful for modeling the explicit solvation around the molecules, locating the faces of the benzene rings in the direction of the protons with the greatest change chemical shift. The size of these spheres agrees with those da Silva et al. reported for organic anions. (da Silva et al., 2009).

**TABLE 1:** Chemical shift changes (ppm) provoked by the aromatic solvent on 1

Proton	$\Delta \delta_{C_{6}D_{6}}$	$\Delta \delta_{C_{7}D_{8}}$
2-6	0.29	0.56
7	0.58	0.85
8	0.35	0.63
9	0.34	0.62
10	0.4	0.67
12	0.21	0.47
11	0.18	0.44
$\Delta \delta_{C_{6}D_{6}}$ = $\delta_{C_{6}D_{6}}$ - $\delta_{CDCl_{3}}$ , $\Delta \delta_{C_{7}D_{8}}$ = $\delta_{C_{7}D_{8}}$ - $\delta_{CDCl_{3}}$		

However, it was not possible to reproduce the experimental chemical shifts in the presence of just the first solvation sphere due to the mobility of solvent molecules on the surface of the solutes. The correlation between experimental and theoretical NMR data in the implicit solvent is *R* ^2^ = 0.98 but when the first solvation sphere is included, the correlation decreases to *R* ^2^ = 0.79. As we will show in the next sections, in spite of the implicit solvent reproducing the NMR shifts, it does not reproduce the energy profiles that agree with the experimental results. Then subsequent solvation spheres need to be included to locate the first sphere in the proper positions on the solute, to reproduce the NMR spectra. We surrounded the solute with its first solvation sphere, with a cluster of solvent molecules. In the case of benzene, we used the cluster reported by Llanio-Trujillo et al., which included 30 benzene molecules. (Llanio-Trujillo et al., 2011) Afterward, we removed benzene molecules until we reached the minimum number of solvent molecules to fit the chemical shift, finding that 22 molecules were enough to achieve a linear fit between the theoretical and experimental data (see S.I. for details). In the case of toluene, we surrounded the solute with the same number of toluene molecules (Frisch et al., 2009), using the Vega ZZ program (Pedretti et al., 2002) to create a solvent cluster. In this case, the correlation between experimental and theoretical NMR data is *R* ^2^ = 0.93; if the implicit solvent is also included, *R* ^2^ = 0.95. da Silva et al. suggested that when enough solvent molecules are added to saturate the solvation shell, the calculated solvation energies should converge. (da Silva et al., 2009) Unlike implicit methods to calculate the cavitation, where a volume is determined, the explicit methods must find the first solvent sphere which directly interacts with the solute and the external shell that maintains the FSS in the right place.

In fact, the number of molecules in the external solvent sphere can not be determined exactly but we have calorimetric evidence to support a similar number of solvent molecules in these solvation spheres. From the calorimetric experiments (data summarized in Table 2), we found that the amount of energy associated with the titration of chloroform over toluene or benzene is small and exothermic. However, when 1 is solved in chloroform and it is titrated over toluene or benzene, the reaction is very exothermic. Using Eq. 1, Δ_*trans*_ *H* = -206.712 kJ/mol for the transfer of 1, from chloroform to benzene whereas from the theoretical calculations with 22 benzene molecules, Δ_*trans*_ *H* is -194.177 kJ/mol, which is very similar considering the size of the system. For the transfer to toluene, Δ_*trans*_ *H* is -539.157 kJ/mol, which involves a larger number of toluene molecules than in the case of benzene. However, we have to note that our interest is not to characterize the external solvent sphere but just to find the solvent environment that allows us to fix the first solvent sphere in the proper positions around the solute to understand the reaction process. From this information, we extrapolated the solvation of the anionic intermediate, the transition states, and cyclic products under the hypothesis that the solvent molecules in the first solvation sphere would remain approximately in the same regions throughout the reaction process.

**TABLE 2:** Heats of mixing, Δ_*trans*_ *H*, when transferring **1** dissolved in chloroform to benzene or toluene.

Titration	Δ*H* (kJ/mole)
Chloroform titrated over toluene	−1.697 ± 0.065
Chloroform titrated over benzene	−0.163 ± 0.046
1 solved in chloroform titrated over toluene	−540.854
1 solved in chloroform titrated over benzene	−206.875 ± 24.158

### 3.2 Solvation energy

**FIGURE 3:**
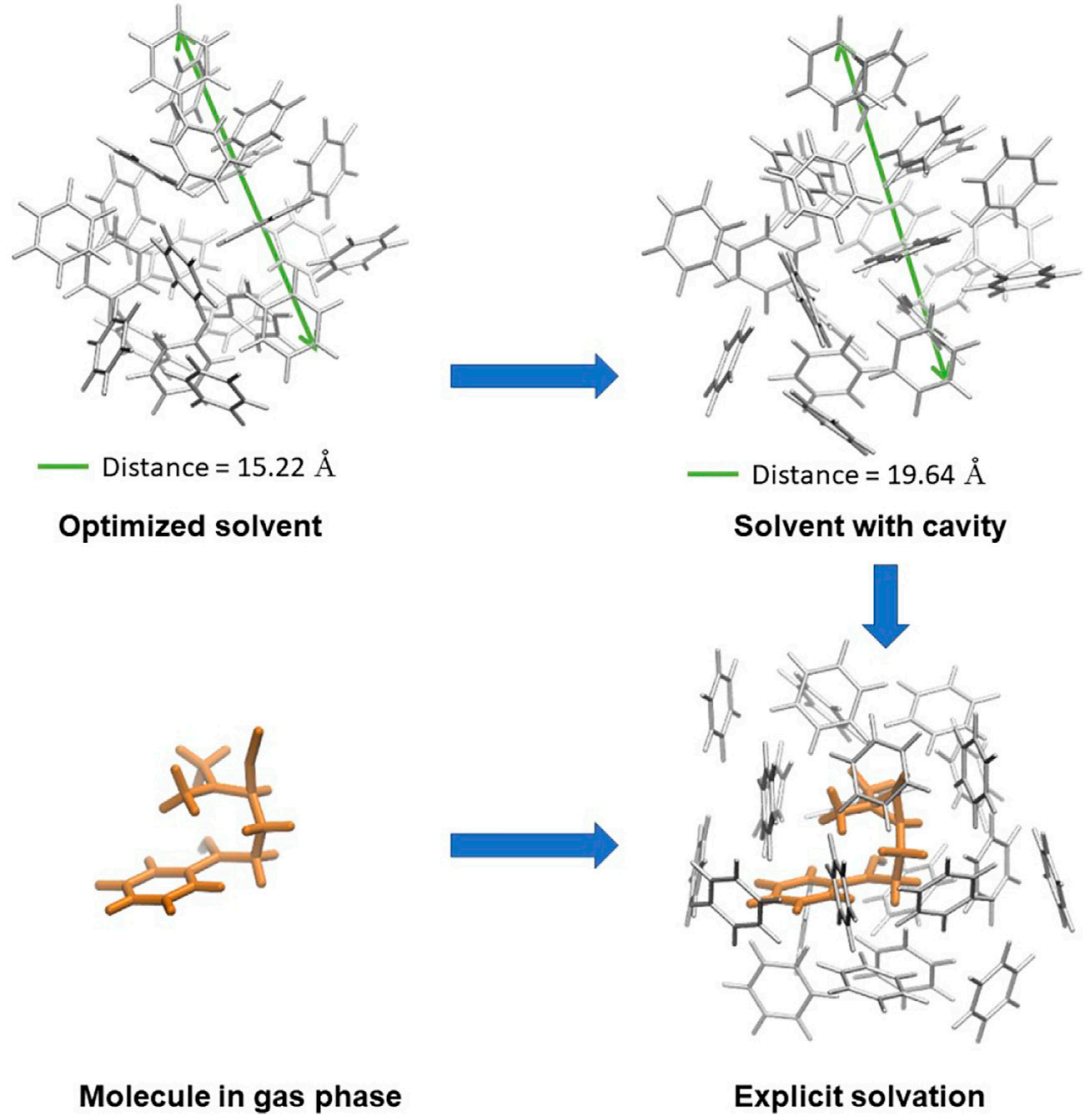
Approach to the solvation energy, which includes the solvent cavity formation and the solute energy change from the gas phase to explicit solvent, counting the specific solvent-solute interactions.

Experimentally, the thermodynamic determination of the heat of solvation takes into account the heat of the broken solute intramolecular interactions and the heat released when each molecule of solute gets in the bulk of the solution and is surrounded by a shell of solvent molecules. (Ramirez-Gualito et al., 2009) Based on the experimental procedure, our approach to the explicit solvation energy accounts for the energies differences associated with the cavity formation and the presence of the solvent interactions with the solute, as shown in Figure 3 and Eq. 2. In this way, the *E*_*solv*_ of **1** in benzene is -379.65 kJ/mol and -516.01 kJ/mol in toluene.

### 3.3 Solvent effect on the conformation

**FIGURE 4:**
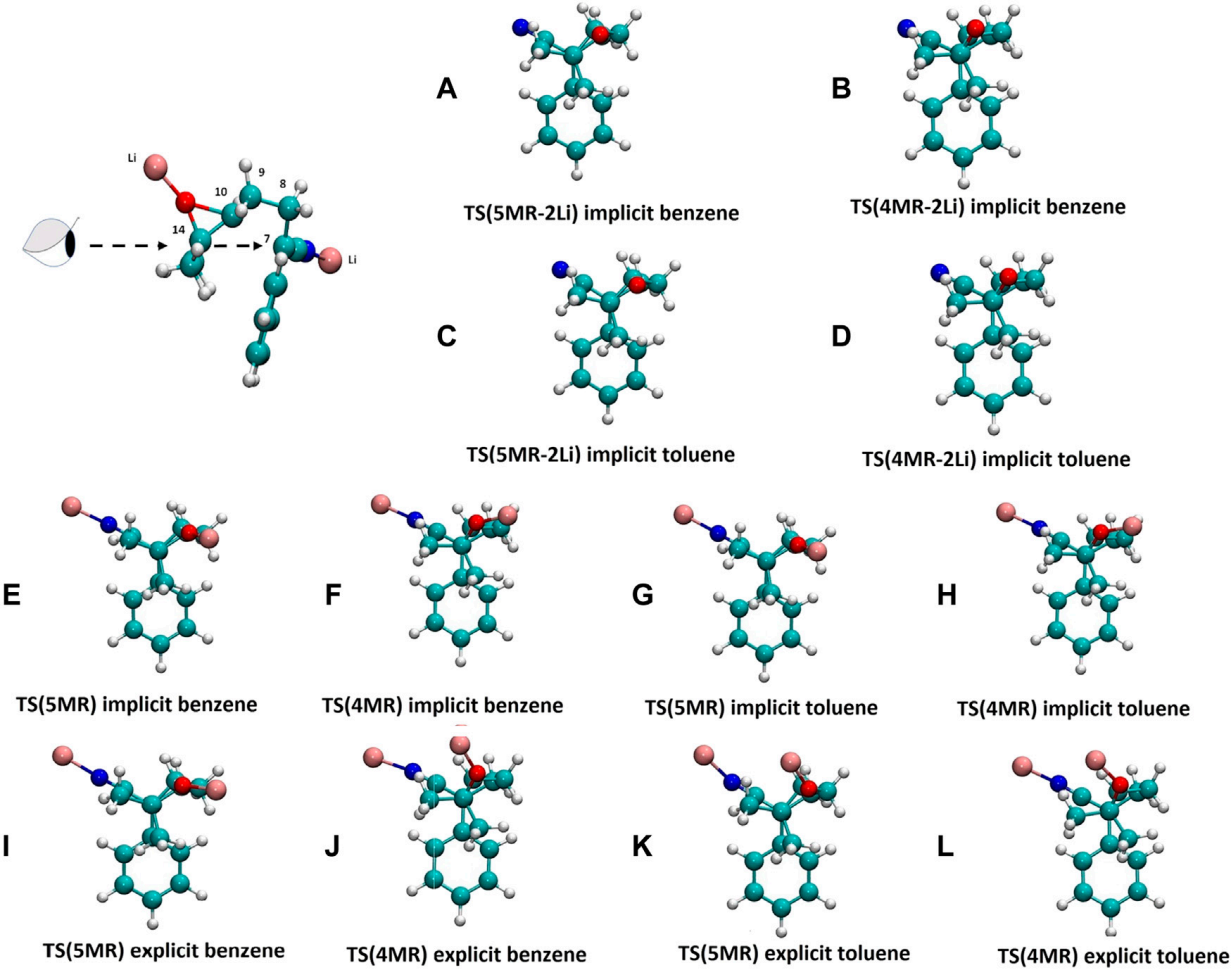
Conformation Newman projections of the transition states in different solvent scenarios. Upper and bottom lines show TS(5MR) and TS(4MR) conformers respectively. In these projections, C_14_ is the proximal atom, while C_7_ is the distal one. **(A)** and **(B)** without cation in implicit benzene; **(C)** and **(D)** without cation in implicit toluene; **(E)** and **(F)** two possible conformation of 1 with two lithium cations in gas phase; **(G)** and **(H)** in gas phase; **(I)** and **(J)** in implicit benzene; **(K)** and **(L)** in implicit toluene; **(M)** and **(N)** in explicit benzene; **(O)** and **(P)** in explicit toluene.

**FIGURE 5:**
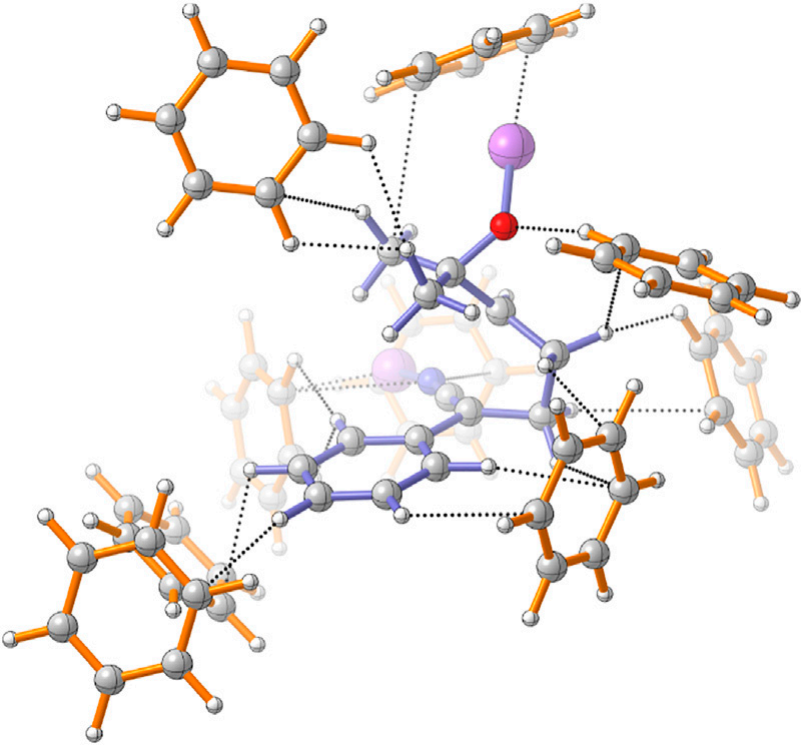
Intermolecular interactions within the benzene solvation sphere of TS(4MR). Four solvent molecules of the second solvation sphere are included.

Usually, the molecular conformation is explained in terms of the stereoelectronic effects (Alabugin, 2016) or by interactions between the molecular and solvent dipoles, (Wang et al., 2014) but there are only a few examples where the specific solute-solvent interactions are taken into account. (Schmitt et al., 2005; Cainelli et al., 2009) In our case, the conformation of 1 changes as the reaction system becomes more complex and realistic. The deprotonation of the *α*-carbon produces a delocalized carbanion with the nitrile group, (Corset et al., 2003; Strzalko et al., 2012; Yang and Fleming, 2017) which attacks the epoxy group in the next step of the reaction. If the carbanion is modeled without a counterion, the conformation allows the negative nitrogen atom and the phenyl ring to be as far as possible. When the counterions are included, one lithium-ion linked to the N-terminus of the nitrile group and the other in the oxygen of the oxirane, the conformation change dramatically by an intramolecular cation-*π* interaction, producing a conformer 104.35 kJ/mol more stable than the open conformer, from which it is not possible to obtain the products, in both gas phase and implicit solvent. The explicit aromatic solvent molecules rectify the conformation (Figure 5), interacting with the lithium atoms and allowing the molecule to assume the orientation where the delocalized carbanion can attack the epoxy group. Figure 4 shows the dihedral angle values of the 4MR and 5MR transition states in the gas phase, implicit and explicit solvents. The solvent presence provokes specific interactions which modify the dihedral angle changing the position of the carbon atom C_7_ and the orientation of C_14_ and C_10_ to give five-membered ring (5MR) and four-membered ring (4MR), respectively. In the gas phase, *TS*(5MR) depicts a dihedral of 10.13°, but the explicit solvation increase it to 13.6° and 18.5° in benzene and toluene, respectively, however, in the implicit solvent, the dihedral change is smaller. In the case of *TS*(4MR), the gas phase dihedral is -30.6° and the implicit solvent increases the value to -28° and - 29.5° in benzene and toluene respectively. The C(7)–C(14) distance is shorter in explicit toluene, whereas the C(7)–C(10) distance is in explicit benzene. These observations correlate with the preference of the reaction in each implicit solvent.

### 3.4 Cyclization reactions within the solvent models

**FIGURE 6:**
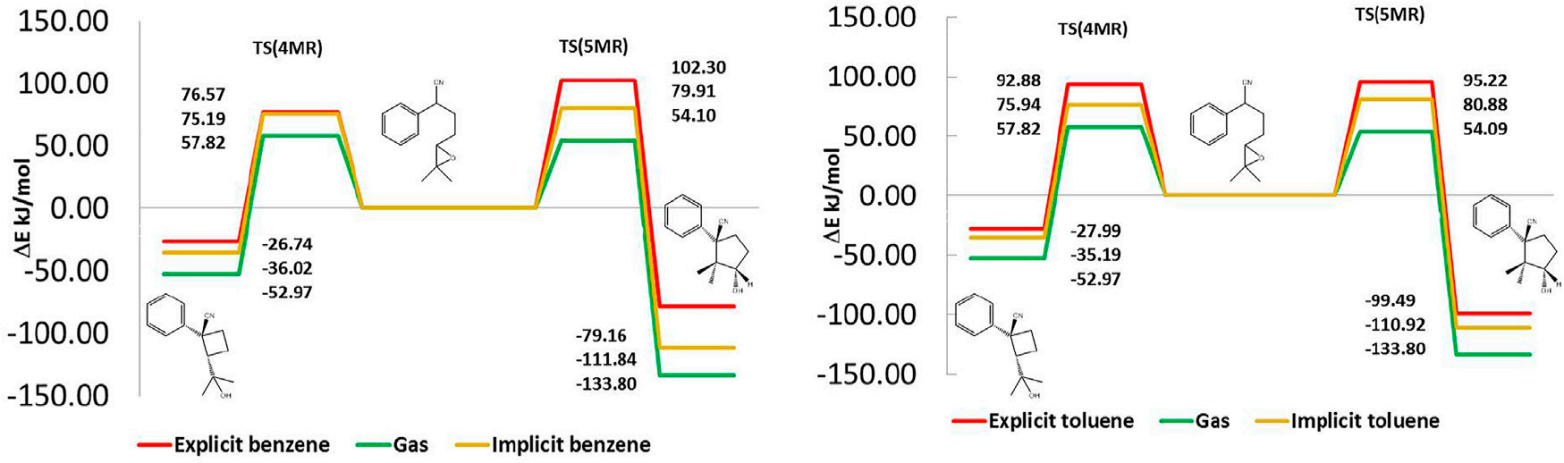
Reaction energy profiles in gas phase, implicit and explicit solvent. Benzene right and Toluene left. In the gas phase and implicit solvent, 5MR is thermodynamically favored and both products present similar reaction barriers. Including explicit solvent reduces the 5MR stability and increases its reaction barrier.

In Figure 6, we present the internal reaction coordinate (IRC) electronic energy profiles of the cyclization of the deprotonated 1 with two lithium-ions linked to the N-extreme of the nitrile group and other in the oxygen of the oxirane. The free energy and enthalpic profiles are presented in the supplementary information. We focused on electronic energy because in the next section this energy is partitioned into solute and solvent contributions, to understand the role of the solvent in the selectivity. This figure shows that in the gas phase, 5MR is thermodynamically more favored than 4MR, whereas both products present similar reaction barriers, with a difference of less than 4.18 kJ/mol. We found the same behavior when the implicit benzene or toluene model is presented. Based on these results, the experimental observation should show just the presence of 5MR. However, the main product is 4MR when benzene is used as a solvent and 5MR with toluene. This contradiction makes clear the necessity to include a better solvent model to improve the description of the cyclization reaction. We found that the inclusion of an explicit solvent is a determinant factor in reproducing the experimental stabilities and barriers. In this way, the observed energetic differences between 5MR and 4MR are linked to specific interactions between them and the solvent.

Figure 6 shows the effect of the explicit solvation on the energetic profile of both cyclization paths. In benzene, the barrier for 5MR is 25.73 kJ/mol larger than for 4MR, whereas in toluene the height of both barriers is almost equal but 5MR is more stable. In this way, 4MR is kinetically favored in benzene, while 5MR is thermodynamically favored in toluene. The presence of the explicit solvent modifies the energetic profile to reproduce the experimental results.

### 3.5 Solvent and solute contribution to the energy

We used the Quantum Theory of Atoms in Molecules (QTAIM) (Bader, 1994) to obtain the solvent (*E*_*solute*_) and solute (*E*_*solvent*_) contributions to the total electronic energy. Each contribution comprises the sum of the atomic energy of every atom in the solvent and the solute. The atomic energies were calculated using the partition of the molecular space defined by the gradient of electron density. Eq. 3 describes the relationship between each contribution and the total energy.

$$E_{\mathrm{total}}=E_{\mathrm{solute}}+E_{\mathrm{solvent}}=\underset{A\in Solute}{\sum }E\left(A\right)+\underset{B\in Solvent}{\sum }E\left(B\right)$$ (3)

**FIGURE 7:**
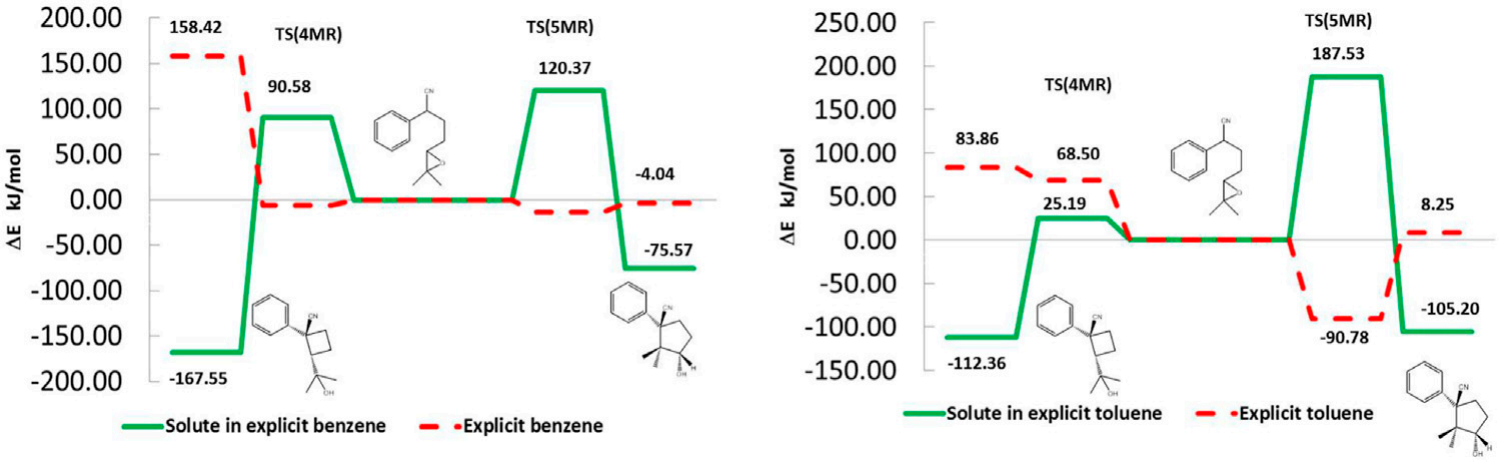
Solute and solvent energetic contributions to the reaction profile in the explicit solvent environment. Benzene right and Toluene left. The solvent modulates the energetic behavior of the solute. In both solvents T5(5MR) is determined by the solute contribution, whereas TS(4MR) is determined by the solute in benzene and by the solvent in toluene.

Figure 7 shows the solute and solvent contributions to the energy of the reactant, transition state, and product. In this way, in benzene, the barrier to 4MR, is originated by the destabilization of 1, by 90.58 kJ/mol, along with a stabilization of the solvent, –6.36 kJ/mol. In the case of toluene for the same product, both contributions are destabilizing, the solvent by 68.49 kJ/mol, and 1 by 25.19 kJ/mol. On the other hand, 5MR destabilizes in both solvents whereas the solvent stabilizes. This behavior is more notorious in the case of toluene. One can observe that the solute in explicit solvent behaves opposite to the molecule in the gas phase or in implicit solvent. The contribution of the solvent modulates the energetic behavior of the solute, which is a determinant to reproduce the experimental observations.

### 3.6 Specific solute-solvent interactions

The analysis of the specific solute-solvent interactions allows us to determine the role of each one in the energy profiles. The interaction energies were estimated with the Espinosa–Molins–Lecomte (EML) approximation. (Espinosa et al., 1998) The EML approximation is based on the value of the potential energy density (*V*(**r**)) at the bond critical points (BCP) that links atoms of the solute with atoms of the solvent molecules. The value of *V*(**r**) is proportional to the interatomic interaction energy. The sum of interaction energies associated with every solute-solvent BCP gives the total interaction energy in which *k* is a constant that depends on each interaction; for hydrogen bonds, the value is 0.5, which was determined by empirical correlations between the interaction energies and BCP properties of a set of hydrogen bonds. In a previous job, our group found, studying the ligand-DNA interaction, that *k* = 0.433 describes non-hydrogen bond weak interactions. (Díaz-Gómez et al., 2017) The energetic contributions of the specific interactions are around 4–29 kJ/mol, table 3 shows the type of weak interactions we found in the systems and the total contribution of each type in each solvent. The experimental facts are supported by the interaction energy contribution since the solute-solvent interactions give a larger stabilization in one of the two solvents, 5MR is more stabilized in toluene, while 4MR is in benzene.

**TABLE 3:** Energetic contribution (kJ/mole) of the specific interactions to the intermolecular interaction energy between transition state structures with the explicit solvent. The times the interaction occurs are in parentheses.

	TS(4MR)		TS(5MR)	
	Benzene	Toluene	Benzene	Toluene
CH⋯*π*	−55.90 (15)	−60.50 (15)	−94.77 (24)	−87.82 (23)
H⋯H	−47.32 (15)	−70.84 (19)	−50.58 (15)	−61.09 (14)
C⋯C	−0.55 (1)	0	0	0
Li⋯C	−70.04 (3)	−27.15 (4)	−27.20 (3)	−36.99 (4)
O⋯H	−30.69 (2)	−15.94 (1)	−42.00 (3)	−24.89 (2)
N⋯X	−11.21 (3)	−18.45 (5)	−13.77 (3)	−21.55 (5)
O⋯X	0	−4.77 (1)	0	0
Total	−217.48 (39)	−196.65 (45)	−228.36 (48)	−230.12 (48)

The number of the specific interactions are in parenthesis.

The breakdown of solute-solvent contributions indicates an important contribution of the specific interactions on the barrier to reaching a transition state. In all cases, the C–H⋯*π* interactions are present in greater numbers, which is to be expected due to the aromatic nature of the solvents, so they have a significant stabilizing contribution. Subsequently, the hydrogen-hydrogen bonds are presented in an average of 16 interactions and represent a net stabilizing contribution. (Matta et al., 2003) In benzene, Li⋯C interactions favor TS(5MR), whereas O⋯H does for TS(4MR).

## 4 Conclusion

In this work, we answered one fundamental question in chemical reactivity, how many solvent molecules should be added and where they should be placed to understand the role of the explicit solvent in the reaction selectivity, in the specific case of the cyclization of *δ*-epoxy nitriles. For this purpose, we performed the experimental determination of the first solvent sphere around a molecule using NMR methods. We found that an epoxy nitrile is surrounded by five benzene or four toluene molecules. With this experimental information, we determined the role of the subsequent solvent spheres ( ∼20 solvent molecules) in locating the first sphere around the solute to reproduce the NMR spectra. We analyzed the specific interactions between the solute with the solvent spheres and how they control the conformation of the reactant and reaction barriers. We showed that explicit solvation is necessary to reproduce experimental results of the cyclization reaction. Also, a new approach to solvation energy is presented, which includes solvent cavity formation and specific solute-solvent interactions from explicit solvation.

## Data Availability

The original contributions presented in the study are included in the article/Supplementary Material, further inquiries can be directed to the corresponding author.
