# Supplementary material for: How solvent determines the molecular reactive conformation and the selectivity: Solvation spheres and energy
Source: Front Chem. 2022 Sep 29;10:1012769. doi: 10.3389/fchem.2022.1012769 (PMC9557062; doi:10.3389/fchem.2022.1012769)
Supplement: Supplementary file 1 [file DataSheet1.PDF]

# Supplementary Material

## 1 SUPPLEMENTARY DATA

### 1.1 General information

Flash chromatography was carried out on handpacked columns of silica gel 60 (230-400 mesh). For thin layer chromatography Merck TLC silica gel 60 F254 aluminum plates were used. HMDSLi was used as a standard commercial solution in toluene from Sigma-Aldrich. Other chemicals were obtained from commercial sources and were used as received. Solvents such as ethyl acetate, acetone, hexane, and dichloromethane, were distilled before being used. Toluene and tetrahydrofuran (THF) were used freshly distilled from metallic sodium. Deuterated solvents were obtained from Cambridge Isotope Laboratories. $^{1}$H-NMR, $^{13}$C-NMR, COSY, HSQC, HMBC, NOESY, and anisotropy titrations, of Epo, C4, and C5, data were obtained using Bruker 750 Avance-III, Bruker 400, and Varian 500. TMS was used as an internal reference.

### 1.2 Synthesis of 5-Iodo-2-methyl-2-pentene, 4-(3,3-dimethyloxiran-2-yl)-2-phenylbutanenitrile and 6-methyl-2-phenylhept-5-enenitrile

5-Iodo-2-methyl-2-pentene was obtained by means of conditions reported for M. Julia and P. Ward.Julia and Ward (1973)

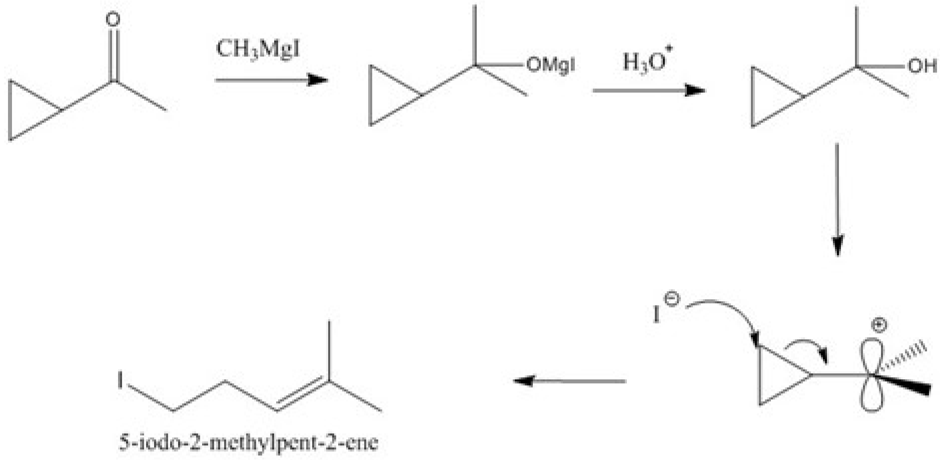


**Figure S1.** Synthesis of 5-Iodo-2-methyl-2-pentene

$^{1}$H-NMR (301 MHz, Chloroform-d) $\delta$ 5.09 (tdq, J = 7.1, 2.9, 1.5 Hz, 1H), 3.10 (t, J = 7.4 Hz, 2H), 2.57 (tdd, J = 8.2, 5.4, 4.4 Hz, 2H), 1.69 (q, J = 1.3 Hz, 4H), 1.65 – 1.57 (m, 3H).

2-phenyl-6-methylhept-5-enonitrile was obtained using conditions reported for W. G. Kofron. Kofron and Hauser (1970)

$^{1}$H-NMR (500 MHz, Chloroform-d): $\delta$ ppm 7.67 - 7.16 (m, 5H), 5.07 (tdt, J = 5.7, 2.8, 1.4 Hz, 1H), 3.78 (dd, J = 8.8, 6.2 Hz, 1H), 2.28 - 2.08 (m, 2H), 2.08 - 1.78 (m, 2H), 1.71 (q, J = 1.3 Hz, 3H), 1.61 (d, J = 1.3

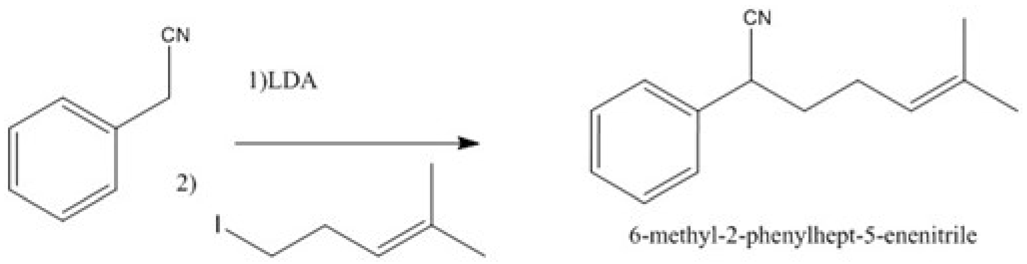


**Figure S2.** Synthesis of 2-phenyl-6-methylhept-5-enonitrile

Hz, 3H). $^{13}$C-NMR (125 MHz, $CDCl_3$): $\delta$ ppm 134.96, 133.80, 129.01(2C), 128.22, 127.31 (2C), 122.01, 119.98, 36.98, 36.01, 25.6, 25.78, 16.99.

4-(3,3-dimethyloxiran-2-yl)-2-phenylbutanenitrile was obtained by means of conditions reported for L. F. Fieser & M. Fieser. Fieser (1967)

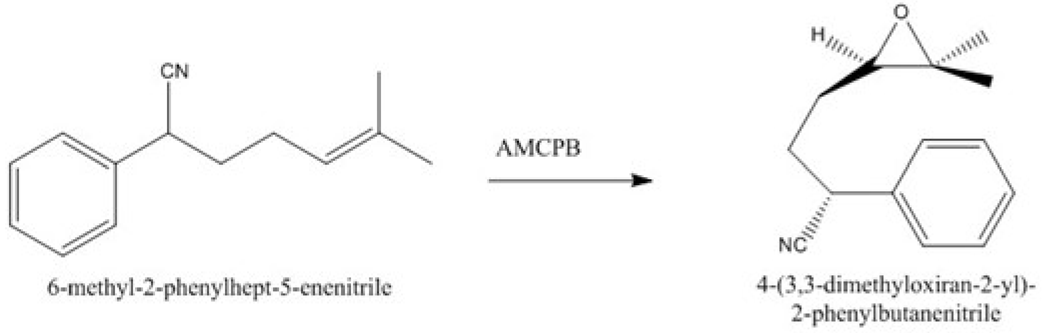


**Figure S3.** Synthesis of 4-(3,3-dimethyloxiran-2-yl)-2-phenylbutanenitrile

$^{1}$H-NMR (750 MHz, Chloroform-d) $\delta$ 7.33 - 7.24 (m, 10H), 3.86 - 3.80 (m, 2H), 2.66 (ddd, J = 14.7, 7.8, 4.7 Hz, 2H), 2.15 - 1.90 (m, 4H), 1.83 - 1.73 (m, 1H), 1.66 (dddd, J = 14.2, 9.5, 6.1, 4.8 Hz, 1H), 1.62 - 1.54 (m, 1H), 1.54 - 1.45 (m, 1H), 1.23 (s, 6H), 1.20 (s, 3H), 1.15 (s, 3H). $^{13}$C-NMR (187.5 MHz, $CDCl_3$) $\delta$ 137.00, 136.68, 130.52 (2C), 130.49 (2C), 129.58, 129.55, 128.76 (2C), 128.53(2C), 121.96, 121.84, 64.71, 64.37, 59.78, 59.67, 38.66, 38.05, 34.62, 34.14, 27.94, 27.23, 26.08, 26.06, 20.09, 20.07.

## 1.3 Synthesis of 2-(2-hydroxypropan-2-yl) 1-phenylcyclobutane-1- carbonitrile and u-3-hydroxy-2,2-dimethyl-1-phenylcyclopentane-1-carbonitrile

2-(2-hydroxypropan-2-yl) 1-phenylcyclobutane-1- carbonitrile and 3-hydroxy-2,2-dimethyl-1-phenylcyclopentane-1-carbonitrile were obtained by means of conditions reported for Montelongo et al. Lujan-Montelongo et al. (2009)

$^{1}$H-NMR (750 MHz, $CDCl_3$ ): $\delta$ ppm 7.61 (dd, J = 7.6, 1.7 Hz, 2H), 7.34 (dt, J = 53.1, 7.6 Hz, 3H), 3.06 (dd, J = 11.0, 8.9 Hz, 1H), 2.73 (ddd, J = 12.4, 10.7, 8.9 Hz, 1H), 2.64 (ddd, J = 12.1, 9.1, 2.6 Hz, 1H), 2.47 (qd, J = 11.0, 9.1 Hz, 1H), 2.12 (dtd, J = 11.5, 8.9, 2.6 Hz, 1H), 1.71 - 1.41 (m, 1H), 1.04 (s, 3H), 0.86 (s, 3H). $^{13}$C-NMR (187.5 MHz, $CDCl_3$ ): $\delta$ ppm 133.58, 128.07 (2C), 127.52, 126.98 (2C), 123.30, 70.55, 54.66, 41.57, 29.02, 26.24, 26.04, 19.21.

3-hydroxy-2,2-dimethyl-1-phenylcyclopentane-1-carbonitrile

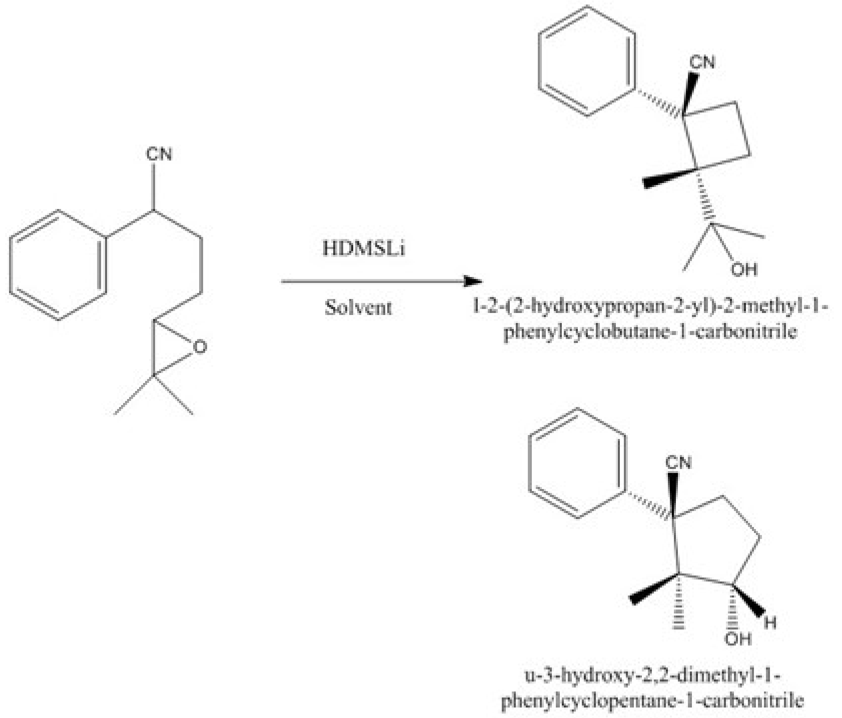


**Figure S4.** Synthesis of 2-(2-hydroxypropan-2-yl) 1-phenylcyclobutane-1- carbonitrile and 3-hydroxy-2,2-dimethyl-1-phenylcyclopentane-1-carbonitrile

$^{1}$ H-NMR (750 MHz, $CDCl_3$): $\delta$ ppm 7.49 (td, J = 7.2, 1.3 Hz, 1H), 7.43 - 7.36 (m, 4H), 4.30 (t, J = 8.4 Hz, 1H), 2.67 (ddd, J = 13.9, 11.9, 6.5 Hz, 1H), 2.38 (dtd, J = 13.6, 9.3, 6.4 Hz, 1H), 2.21 (ddd, J = 13.9, 9.6, 4.2 Hz, 1H), 1.73 - 1.68 (m, 1H), 1.13 (s, 4H), 0.51 (s, 3H). $^{13}$C-NMR (187.5 MHz, $CDCl_3$): $\delta$ ppm 134.99, 128.30(2C), 128.28, 127.68(2C), 123.93, 79.56, 54.22, 49.21, 29.71, 28.98, 21.22, 14.61.

5-hydroxy-5-methyl-1-phenylhexan-1-one

$^{1}$H-NMR (750 MHz, Chloroform-d) $\delta$ 7.97 (dd, J = 8.2, 1.4 Hz, 2H), 7.52 - 7.47 (m, 1H), 7.32 (t, J = 7.8 Hz, 2H), 2.72 (dt, J = 8.1, 4.5 Hz, 1H), 1.72 (dddd, J = 13.4, 9.1, 7.4, 4.3 Hz, 2H), 1.32 (ddd, J = 8.8, 4.8, 3.6 Hz, 1H), 1.25 (d, J = 5.7 Hz, 6H), 1.21 - 1.16 (m, 1H), 1.13 (tddd, J = 15.0, 14.3, 6.0, 3.6, 0.0 Hz, 0H). $^{13}$C-NMR (189 MHz, $CDCl_3$) $\delta$ 200.37, 138.04, 134.99, 127.68, 123.93, 79.56, 77.39, 77.22, 77.05, 68.97, 36.93, 30.64, 29.98, 29.89, 22.48, 15.99.

## 1.4 NMR Spectra

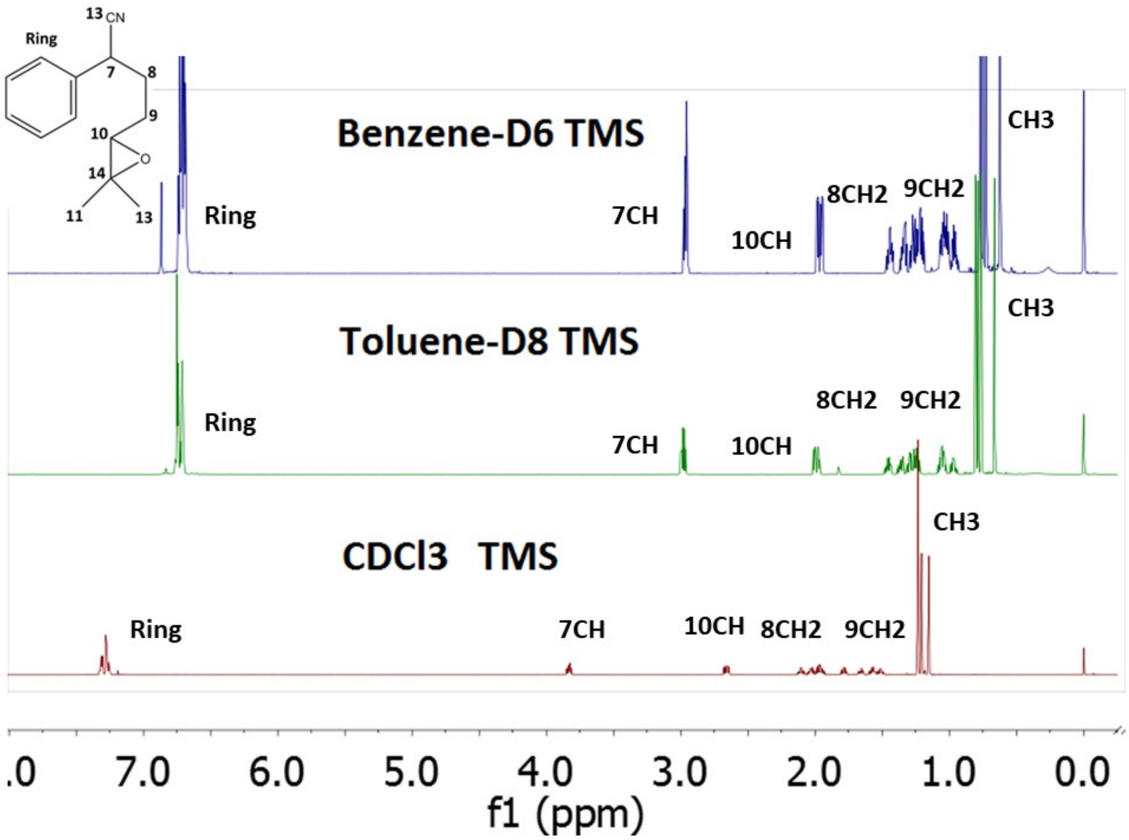


**Figure S5.** $^{1}$H-NMR 4-(3,3-dimethyloxiran-2-yl)-2-phenylbutanenitrile

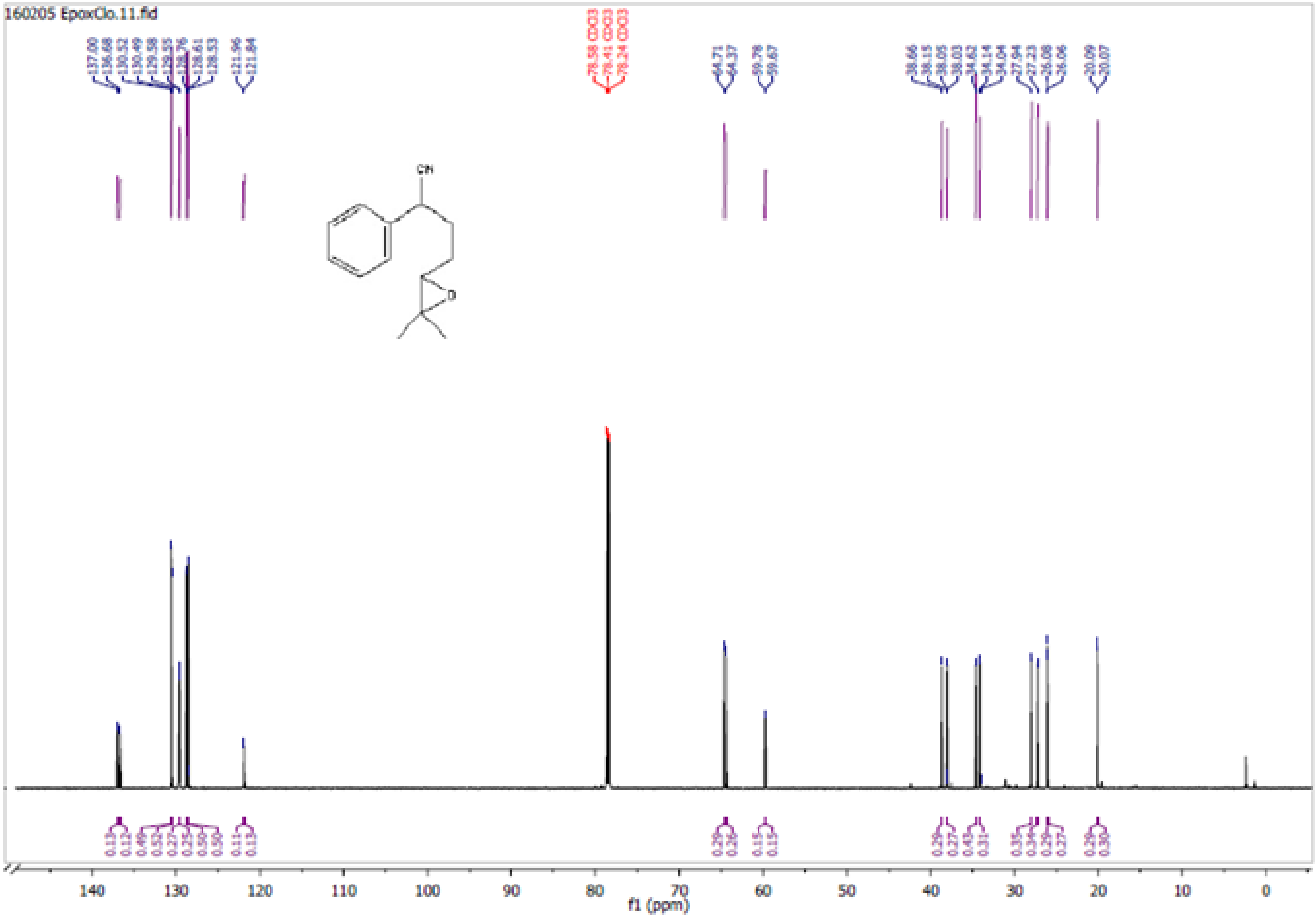

**Figure S6.** $^{13}$C-NMR 4-(3,3-dimethyloxiran-2-yl)-2-phenylbutanenitrile

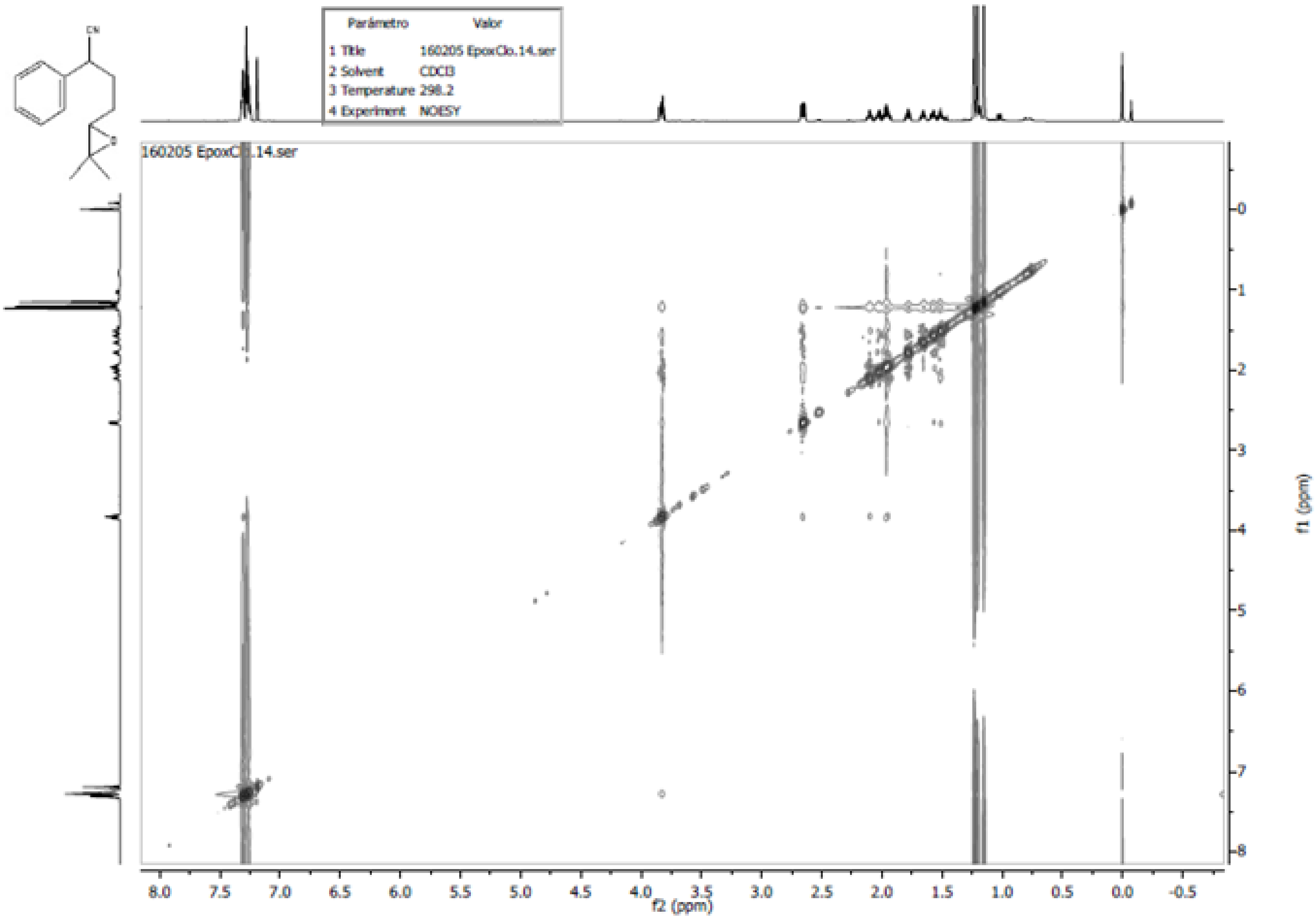


**Figure S7.** NOESY 4-(3,3-dimethyloxiran-2-yl)-2-phenylbutanenitrile

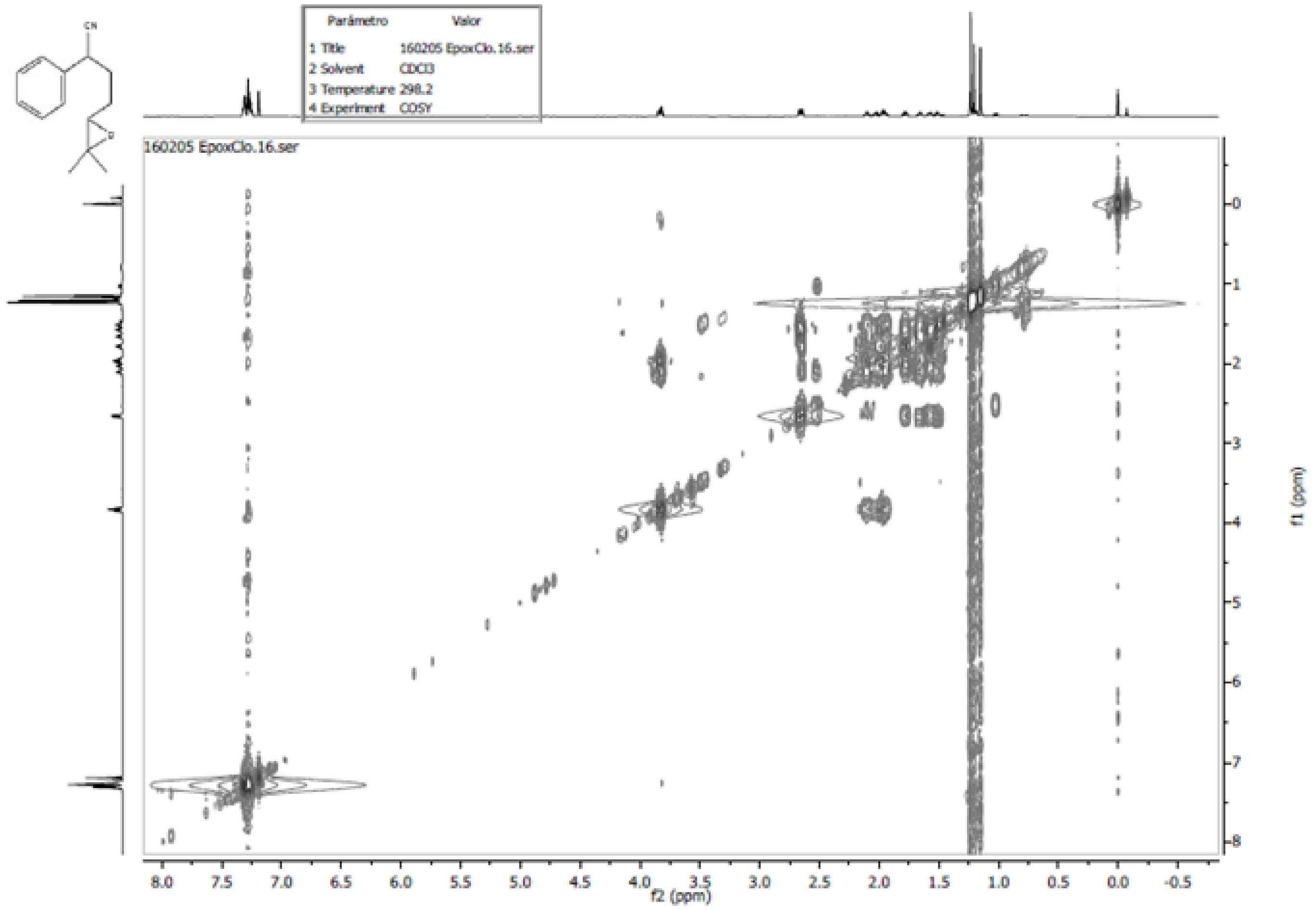


**Figure S8.** COSY 4-(3,3-dimethyloxiran-2-yl)-2-phenylbutanenitrile

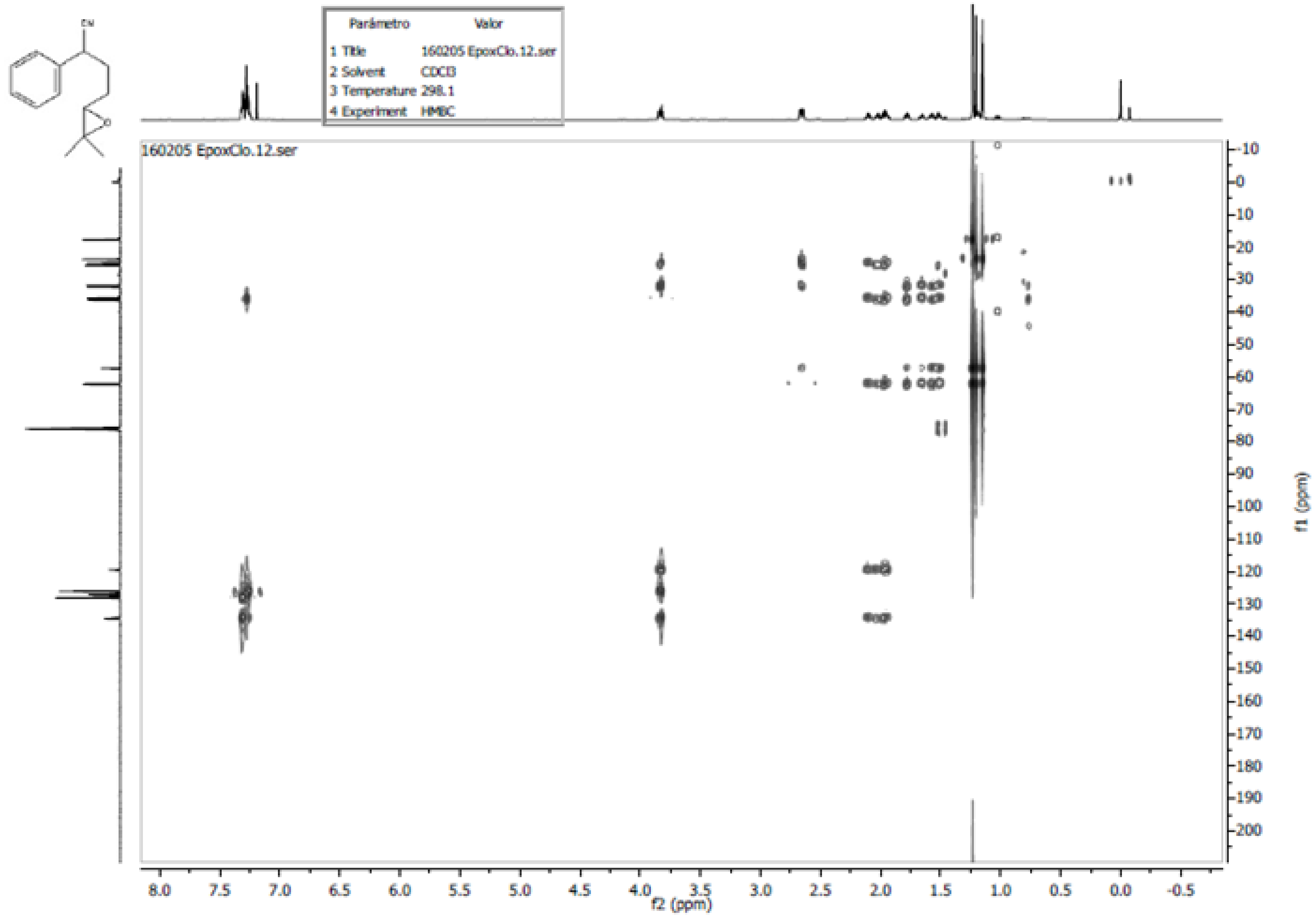


**Figure S9.** HMBC 4-(3,3-dimethyloxiran-2-yl)-2-phenylbutanenitrile

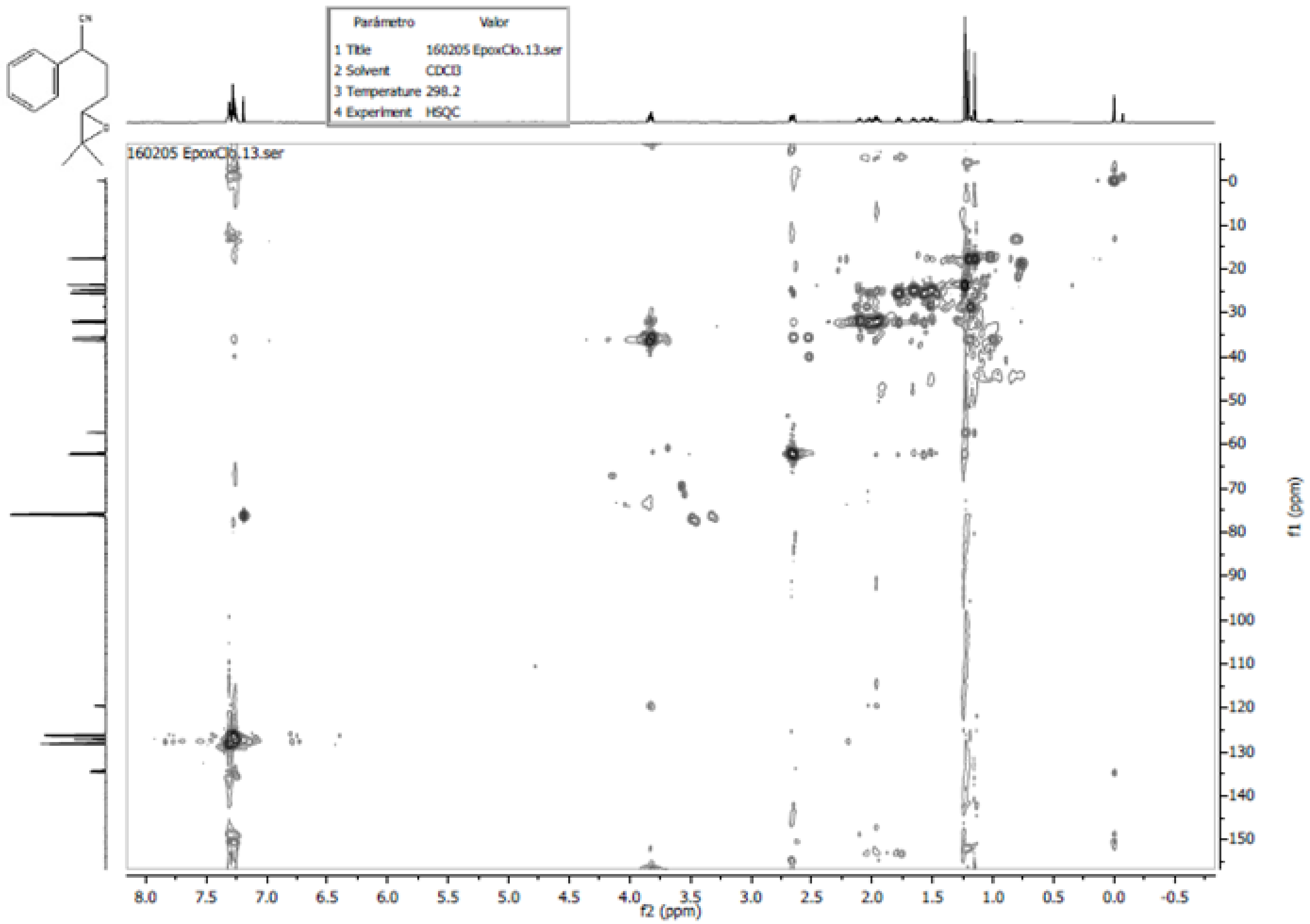


**Figure S10.** HSQC 4-(3,3-dimethyloxiran-2-yl)-2-phenylbutanenitrile

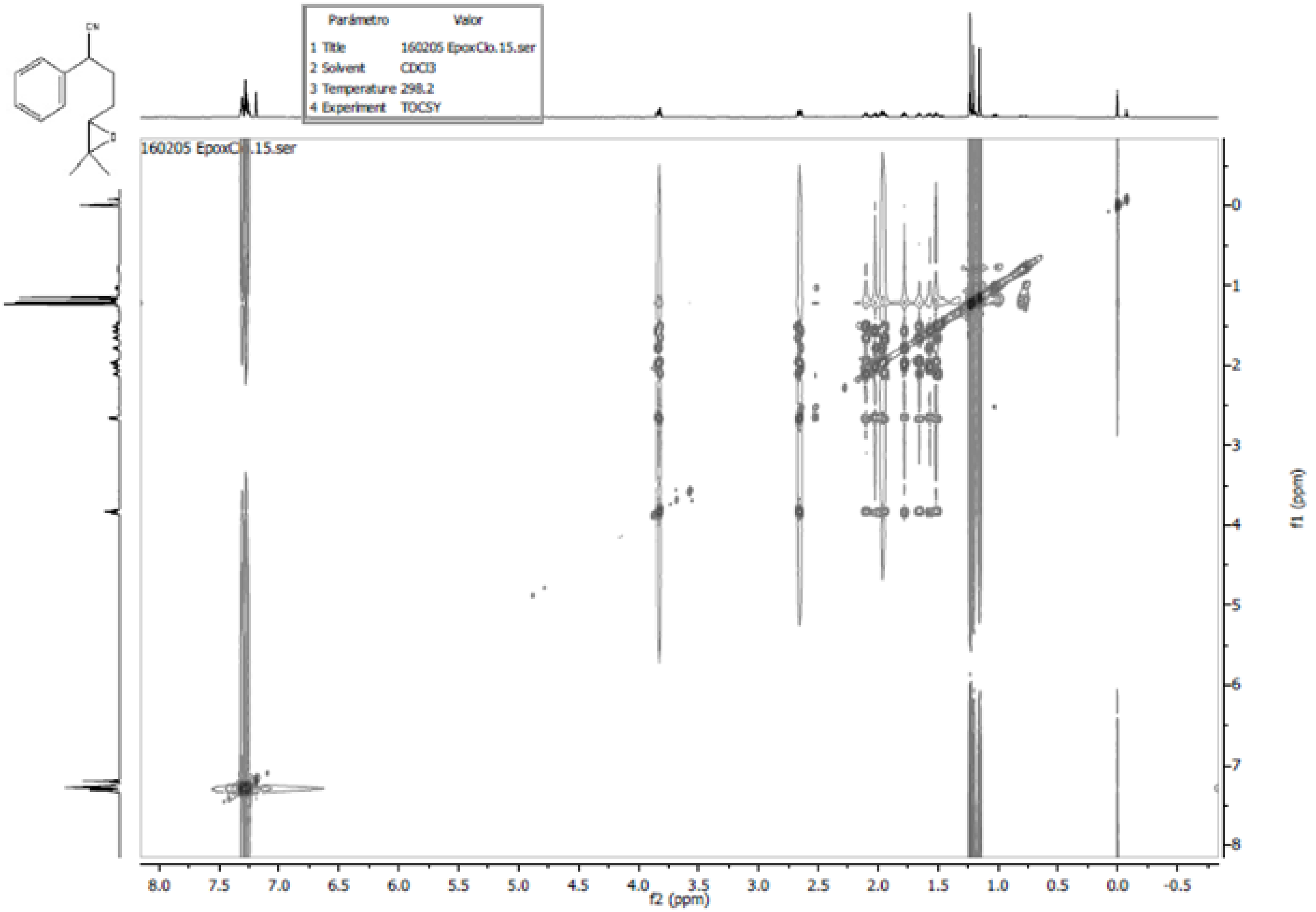


**Figure S11.** TOCSY 4-(3,3-dimethyloxiran-2-yl)-2-phenylbutanenitrile

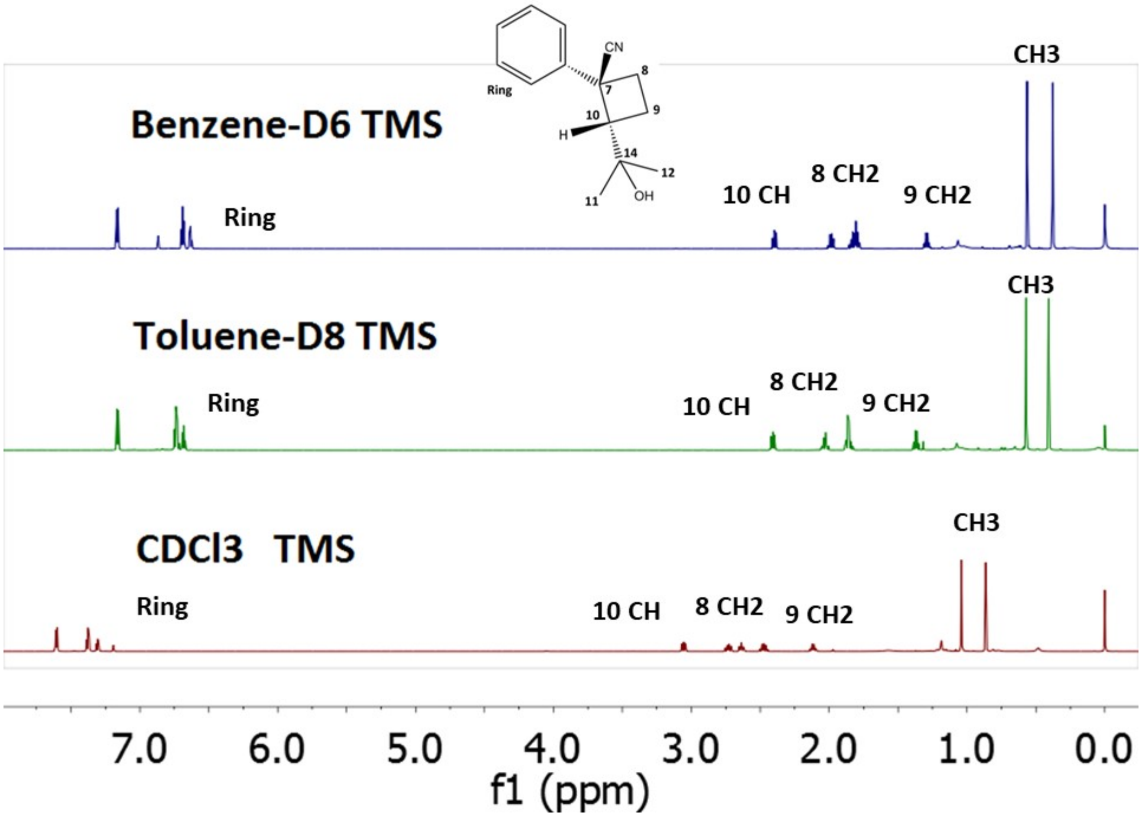


**Figure S12.** $^{1}$H-NMR 2-(2-hydroxypropan-2-yl) 1-phenylcyclobutane-1- carbonitrile

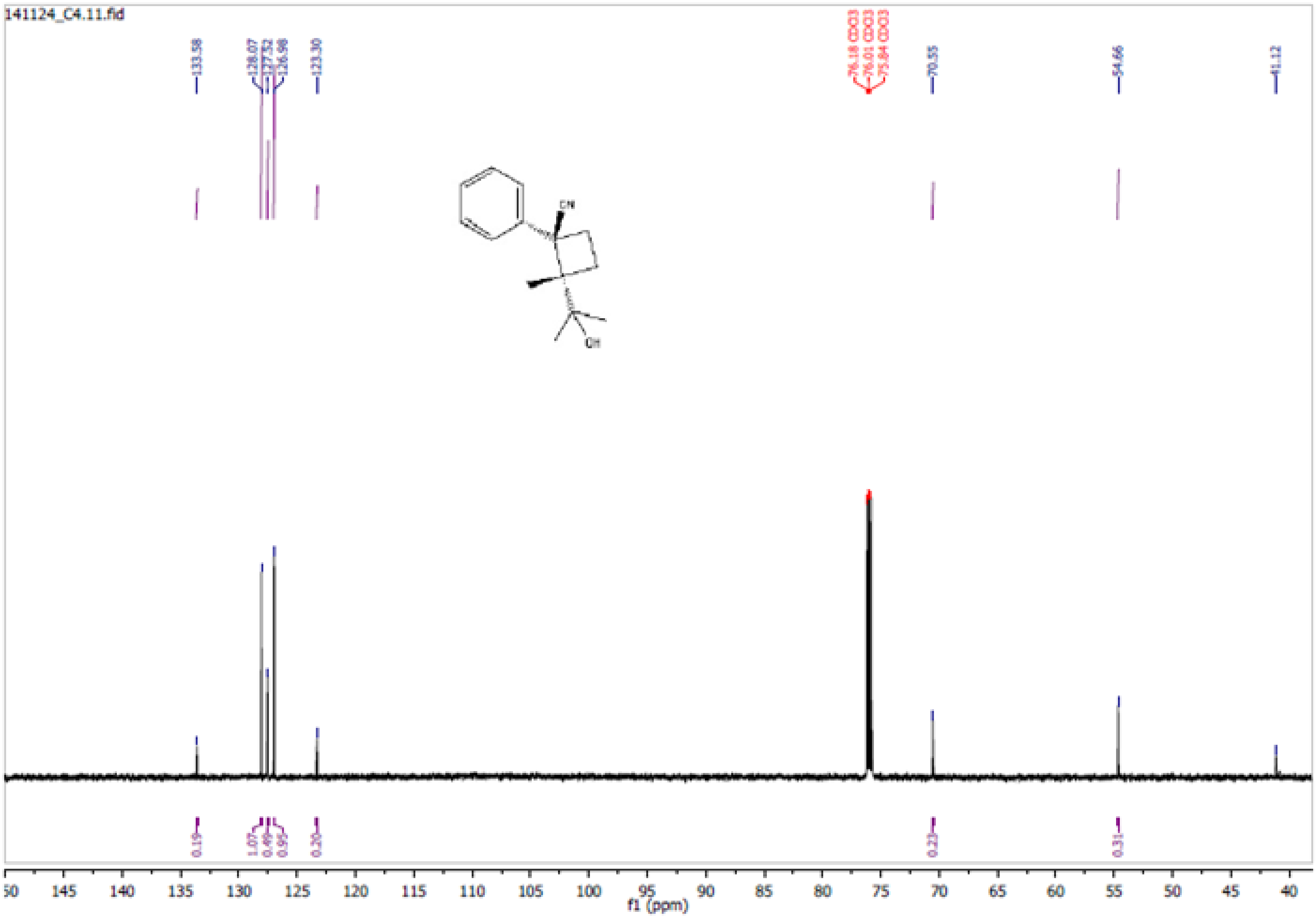


**Figure S13.** $^{13}$C-NMR 2-(2-hydroxypropan-2-yl) 1-phenylcyclobutane-1- carbonitrile

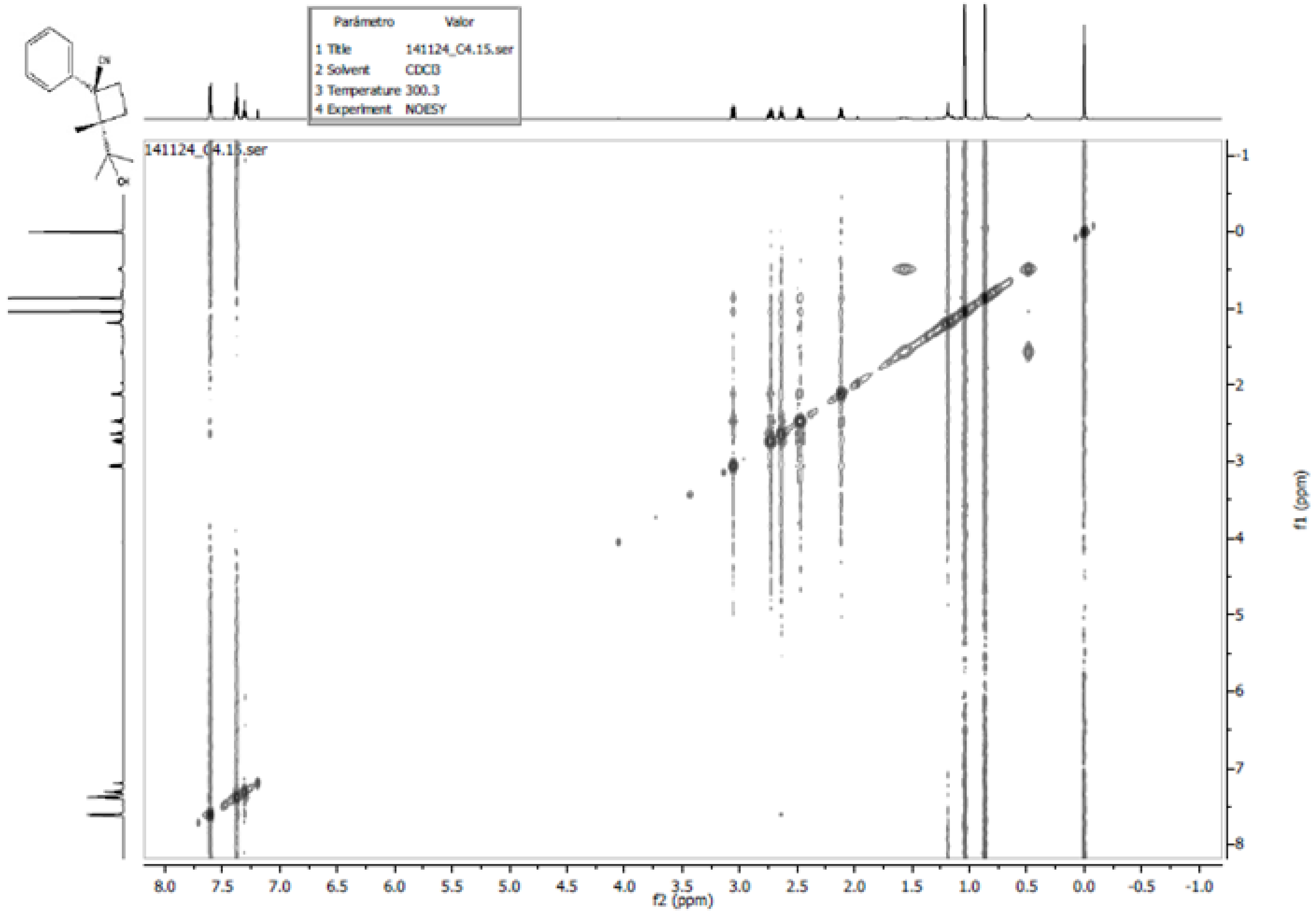


**Figure S14.** NOESY 2-(2-hydroxypropan-2-yl) 1-phenylcyclobutane-1- carbonitrile

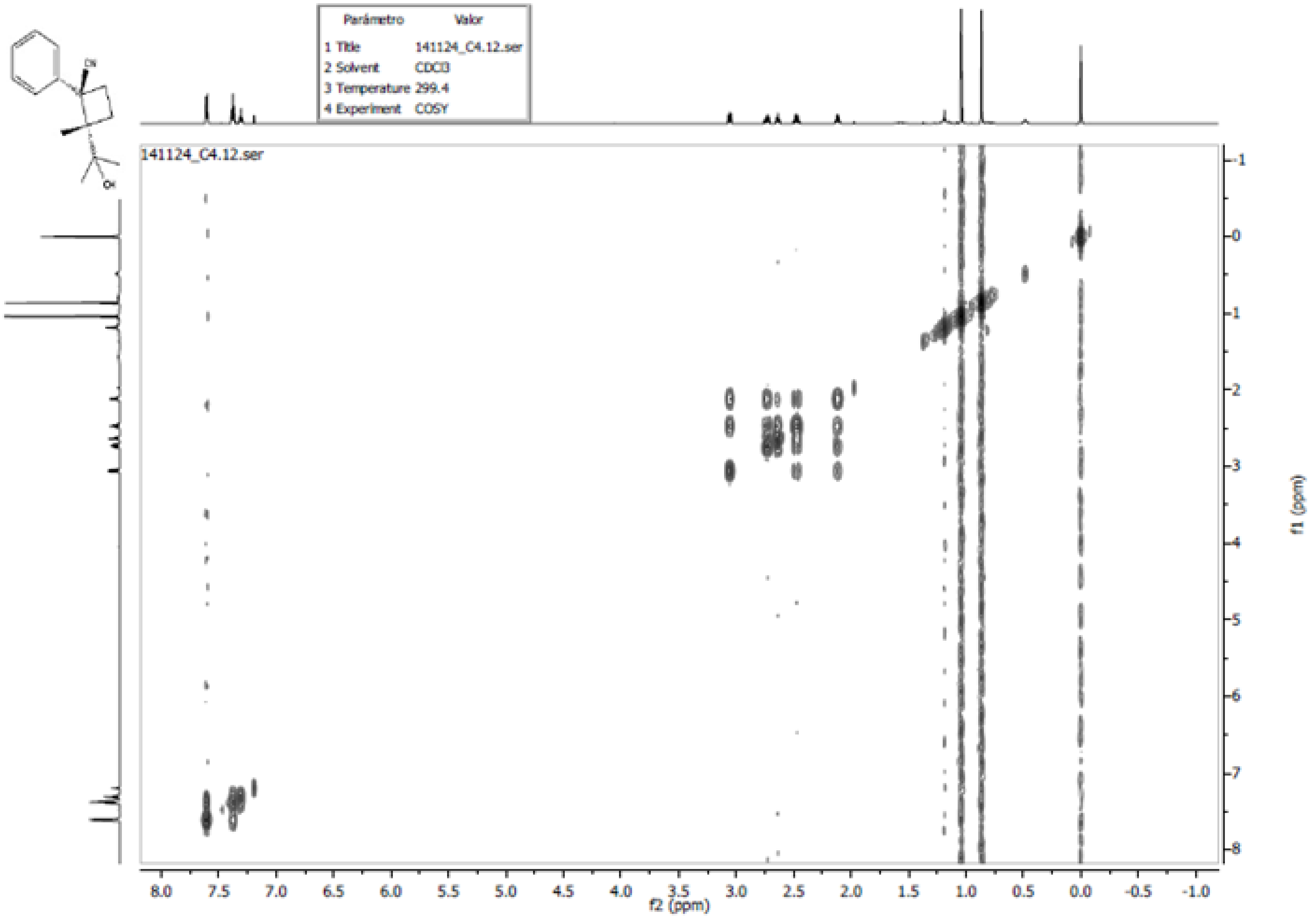


**Figure S15.** COSY 2-(2-hydroxypropan-2-yl) 1-phenylcyclobutane-1- carbonitrile

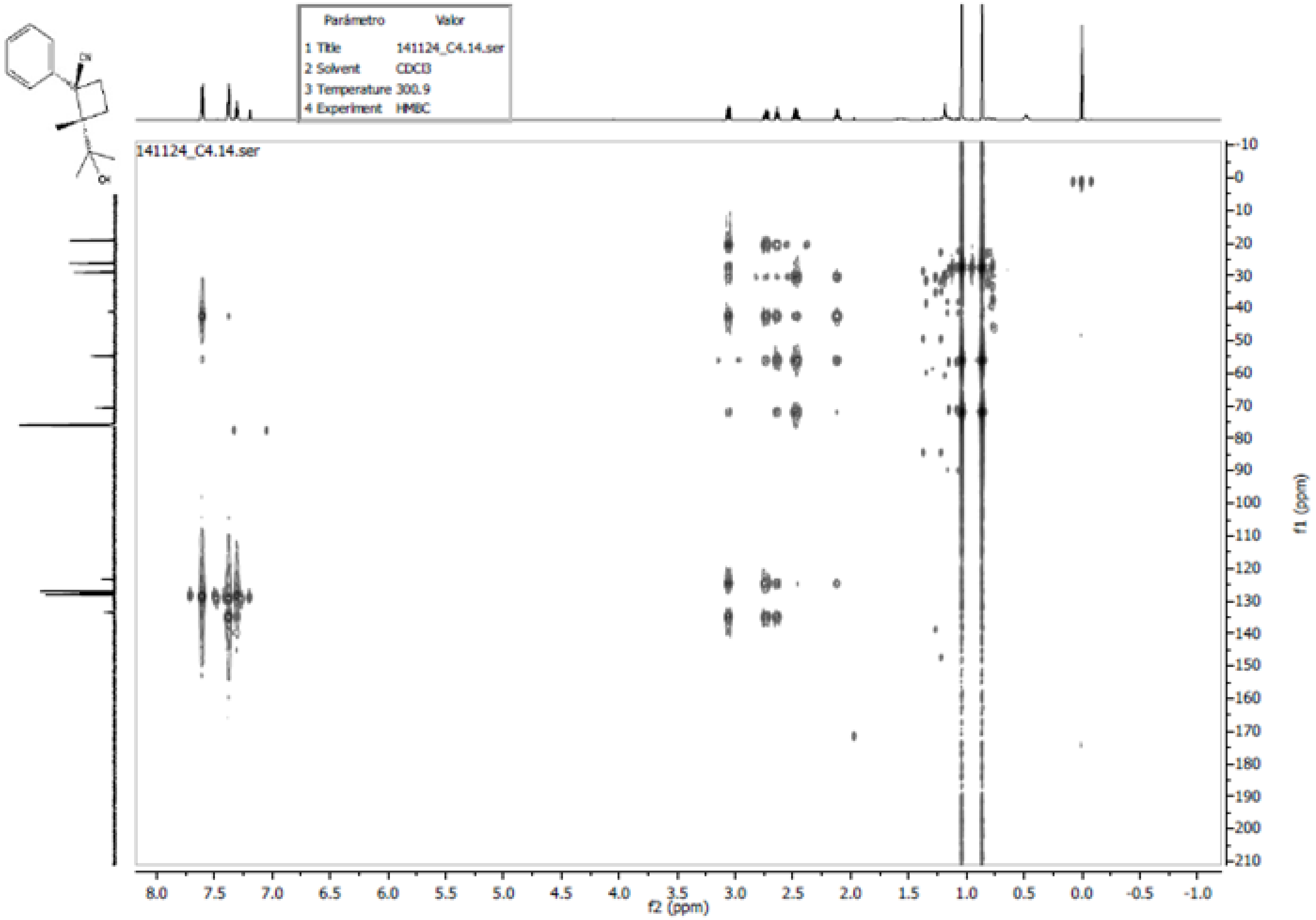


**Figure S16.** HMBC 2-(2-hydroxypropan-2-yl) 1-phenylcyclobutane-1- carbonitrile

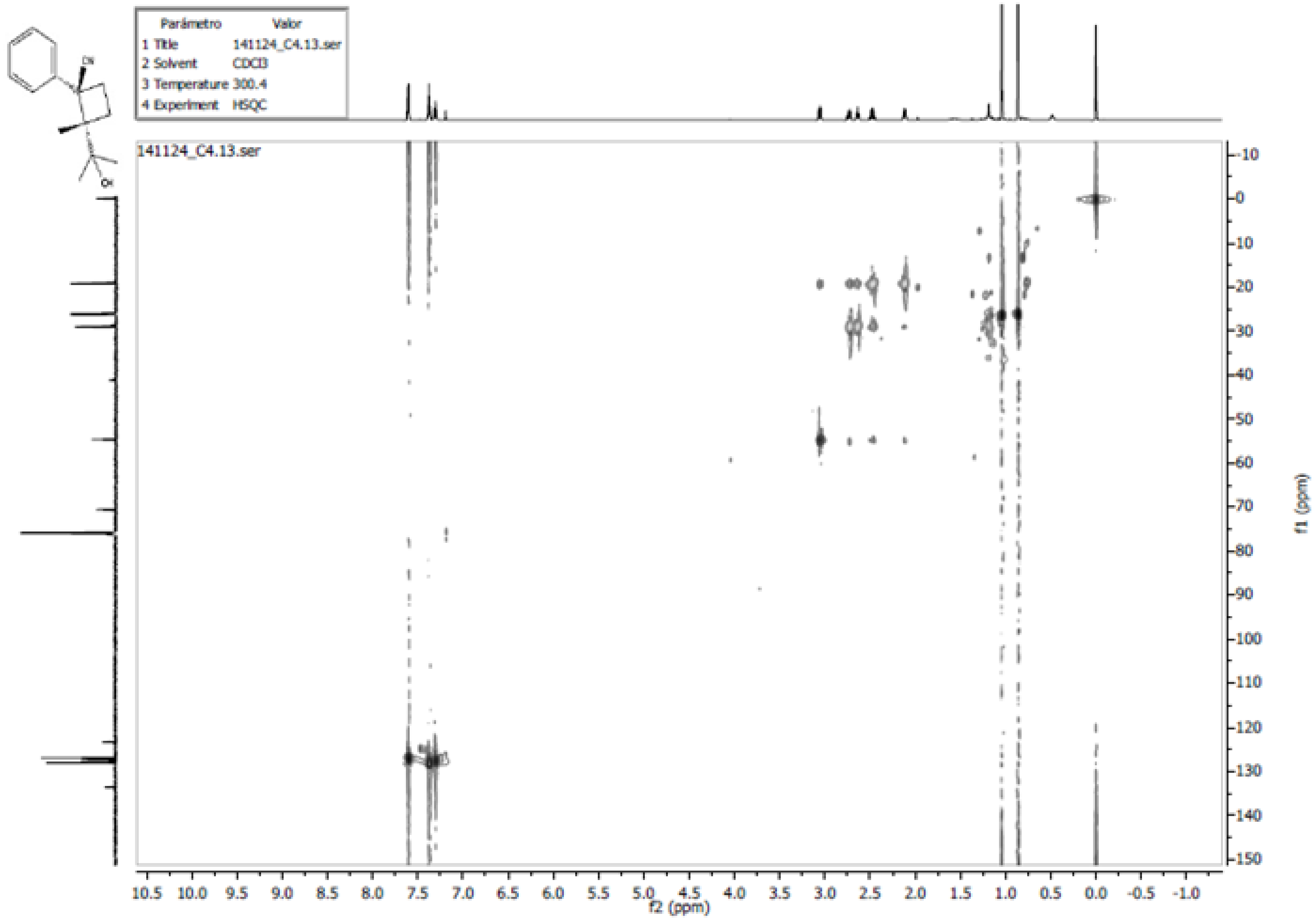


**Figure S17.** HSQC 2-(2-hydroxypropan-2-yl) 1-phenylcyclobutane-1- carbonitrile

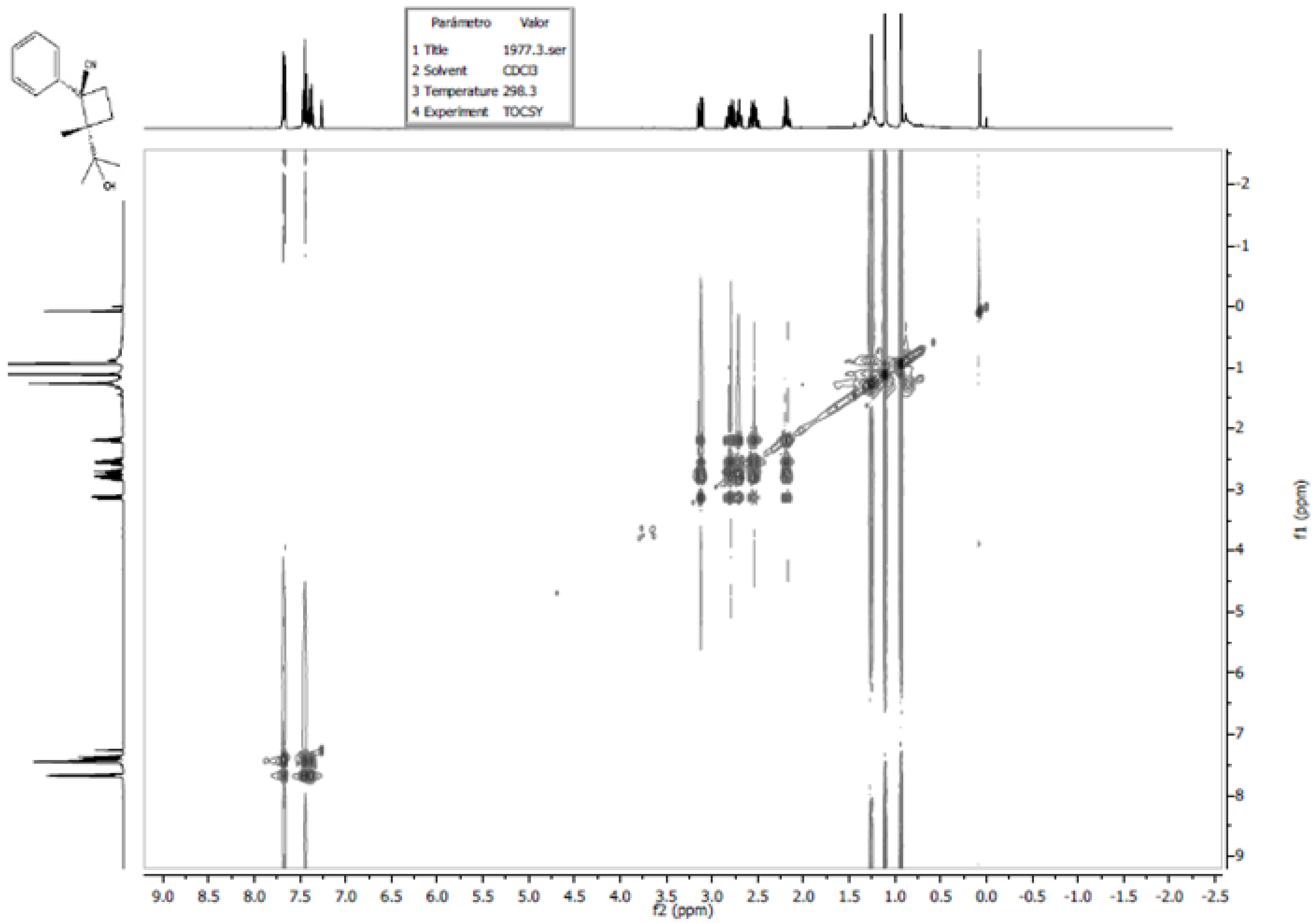


**Figure S18.** TOCSY 2-(2-hydroxypropan-2-yl) 1-phenylcyclobutane-1- carbonitrile

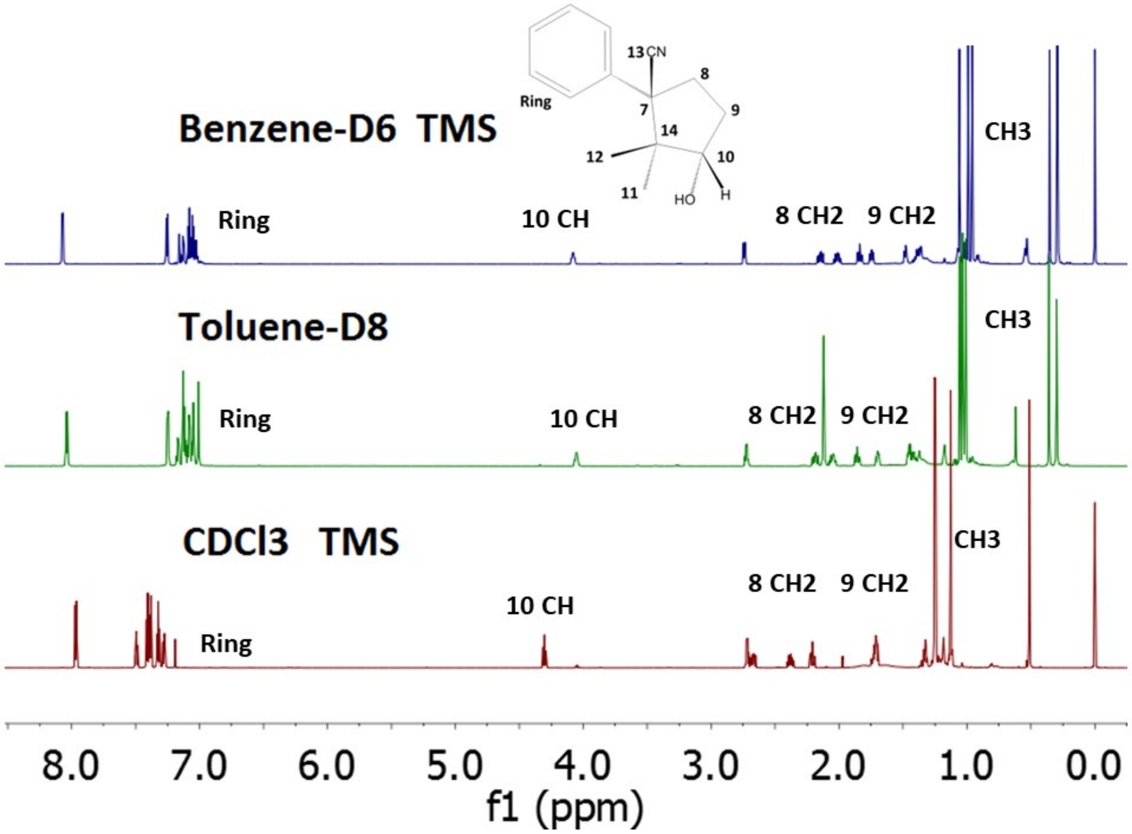


**Figure S19.** $^{1}$H-NMR 3-hydroxy-2,2-dimethyl-1-phenylcyclopentane-1-carbonitrile

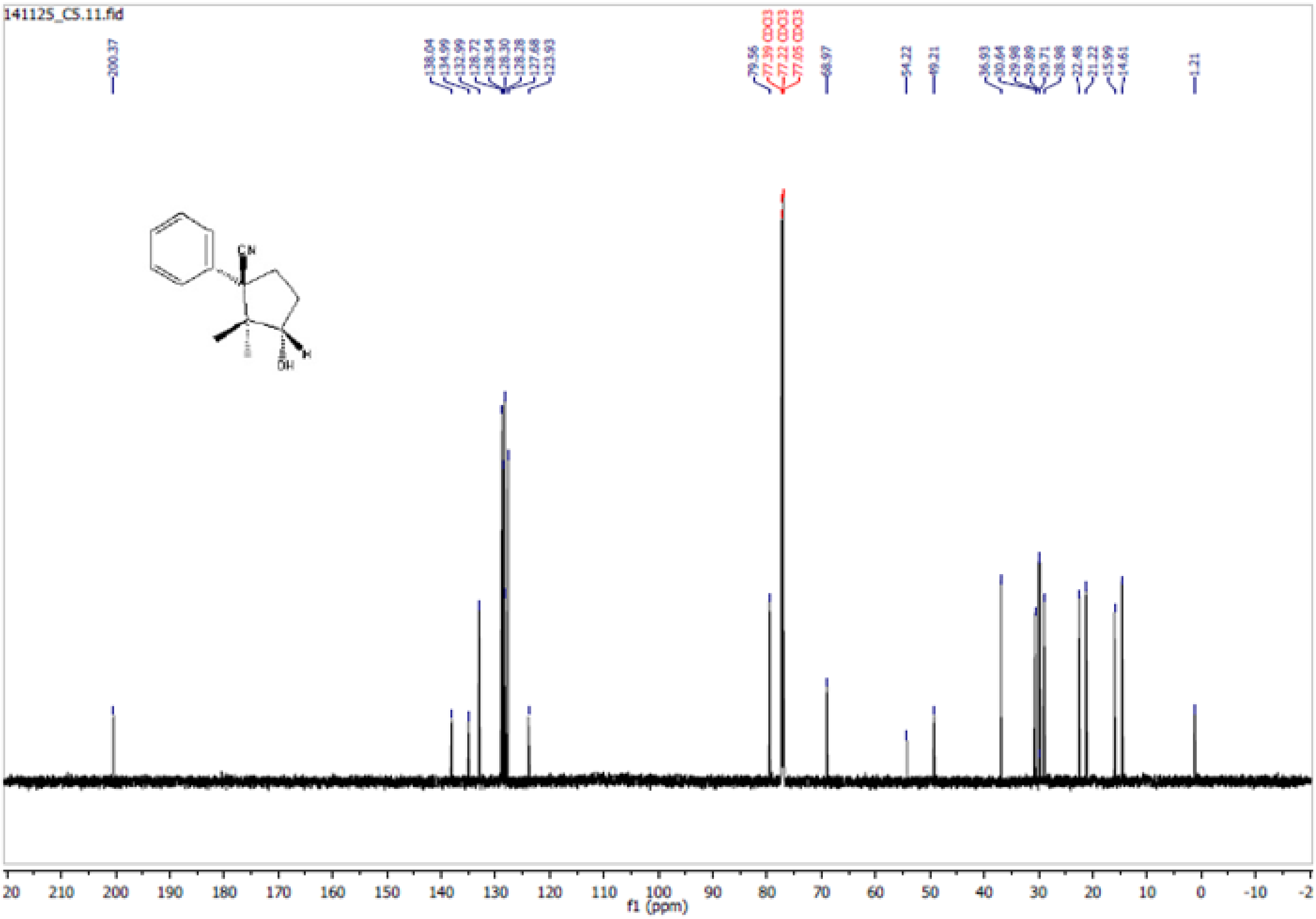


**Figure S20.** $^{13}$C-NMR 3-hydroxy-2,2-dimethyl-1-phenylcyclopentane-1-carbonitrile

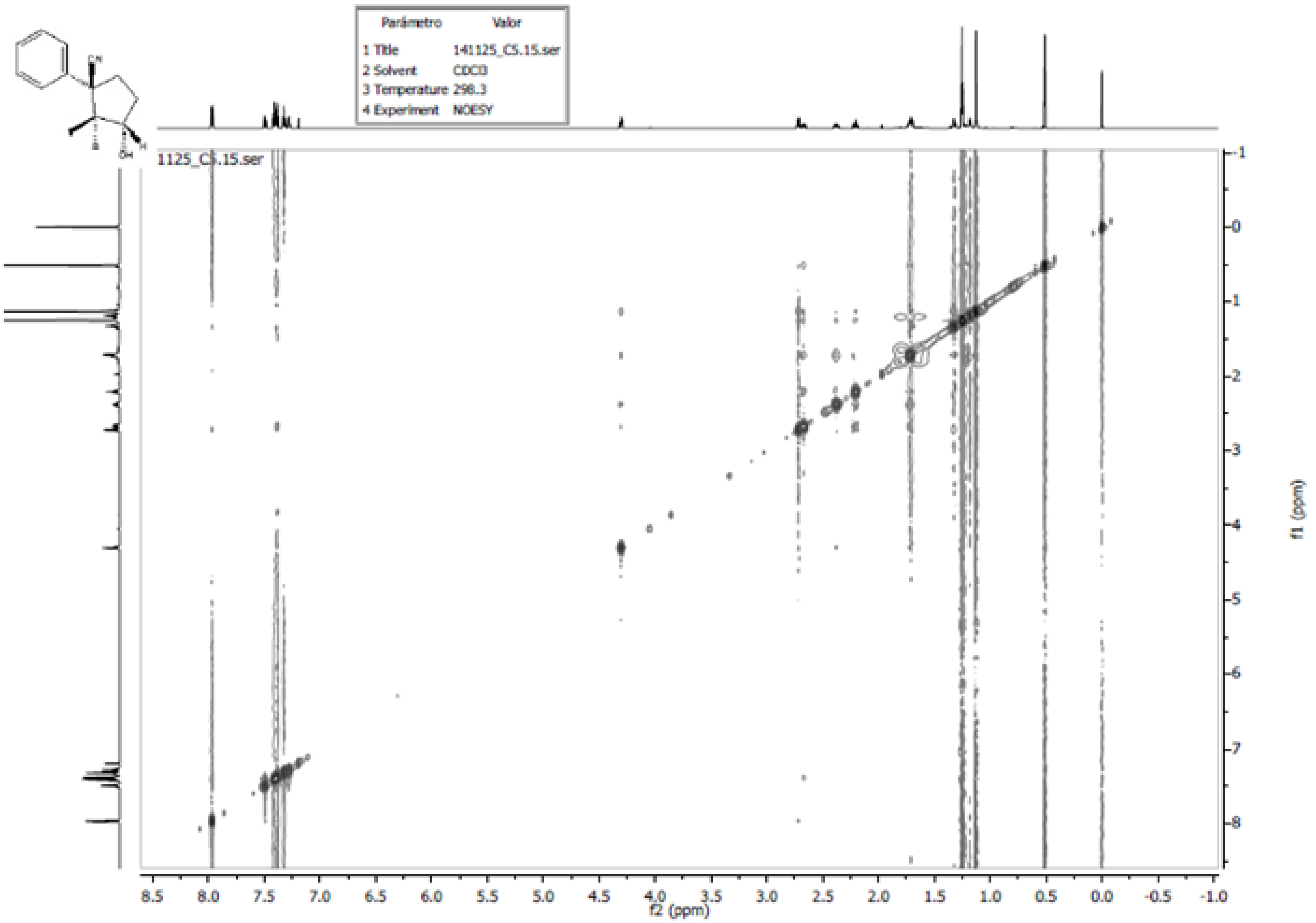


**Figure S21.** NOESY 3-hydroxy-2,2-dimethyl-1-phenylcyclopentane-1-carbonitrile

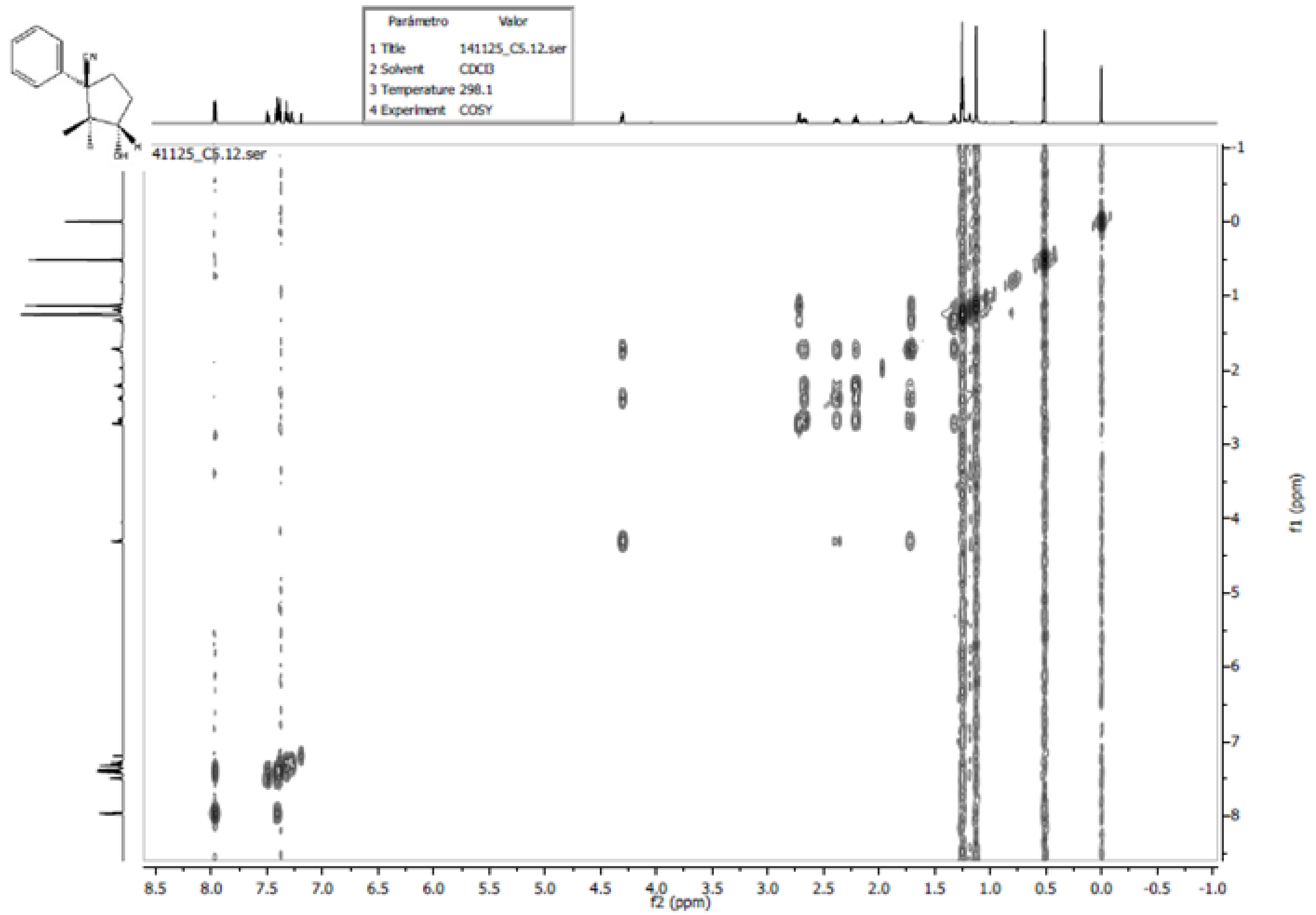


**Figure S22.** COSY 3-hydroxy-2,2-dimethyl-1-phenylcyclopentane-1-carbonitrile

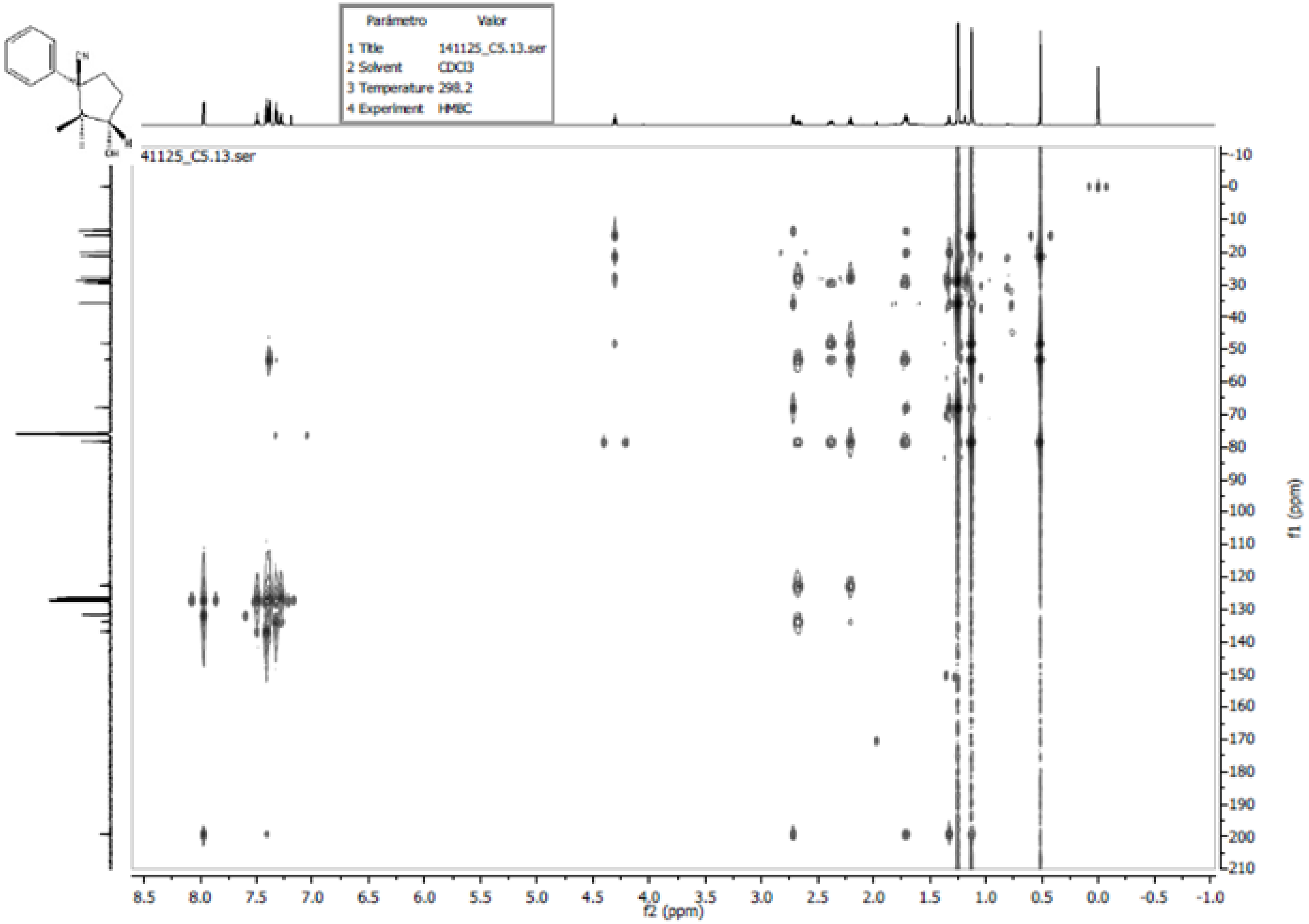


**Figure S23.** HMBC 3-hydroxy-2,2-dimethyl-1-phenylcyclopentane-1-carbonitrile

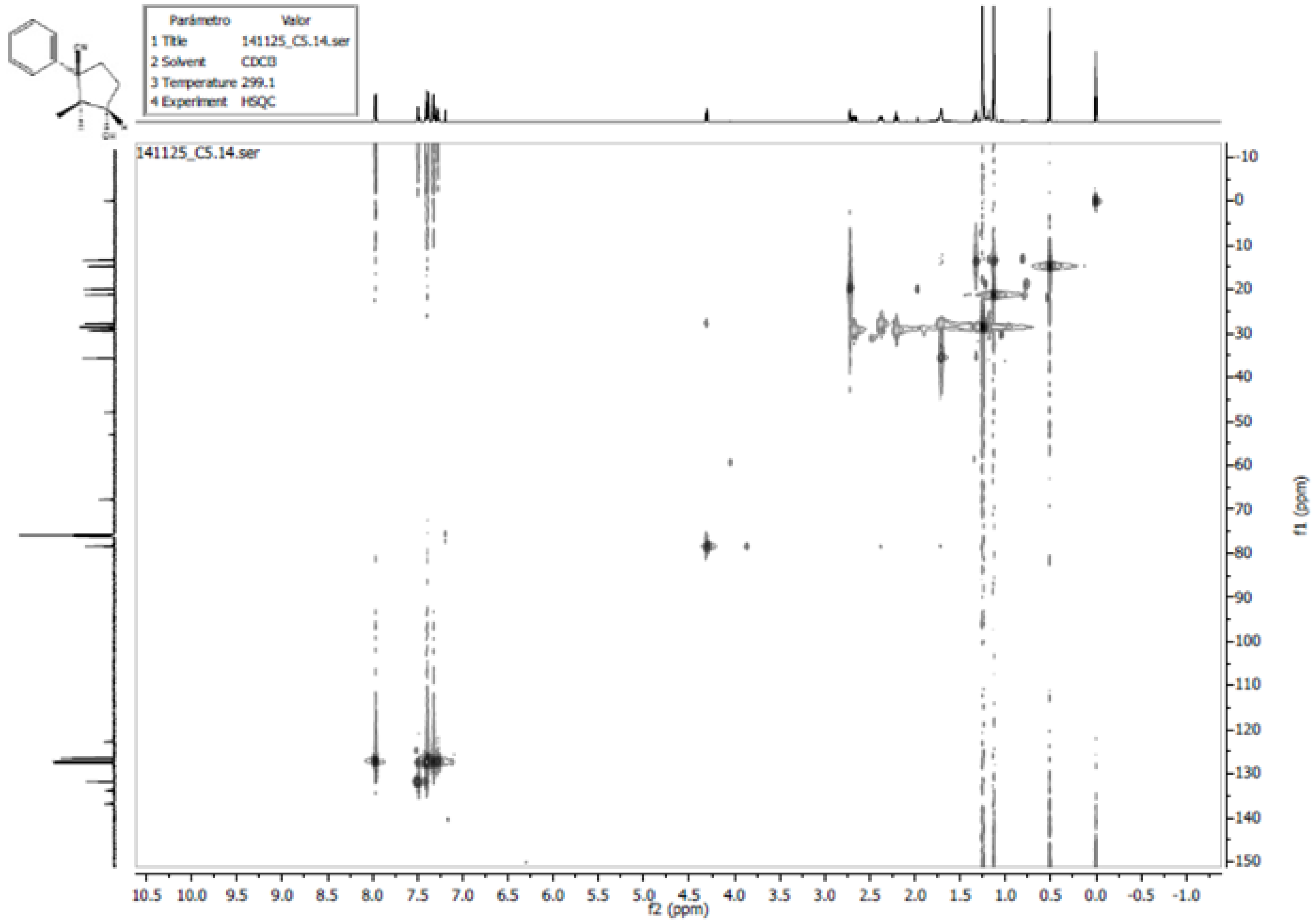


**Figure S24.** HSQC 3-hydroxy-2,2-dimethyl-1-phenylcyclopentane-1-carbonitrile

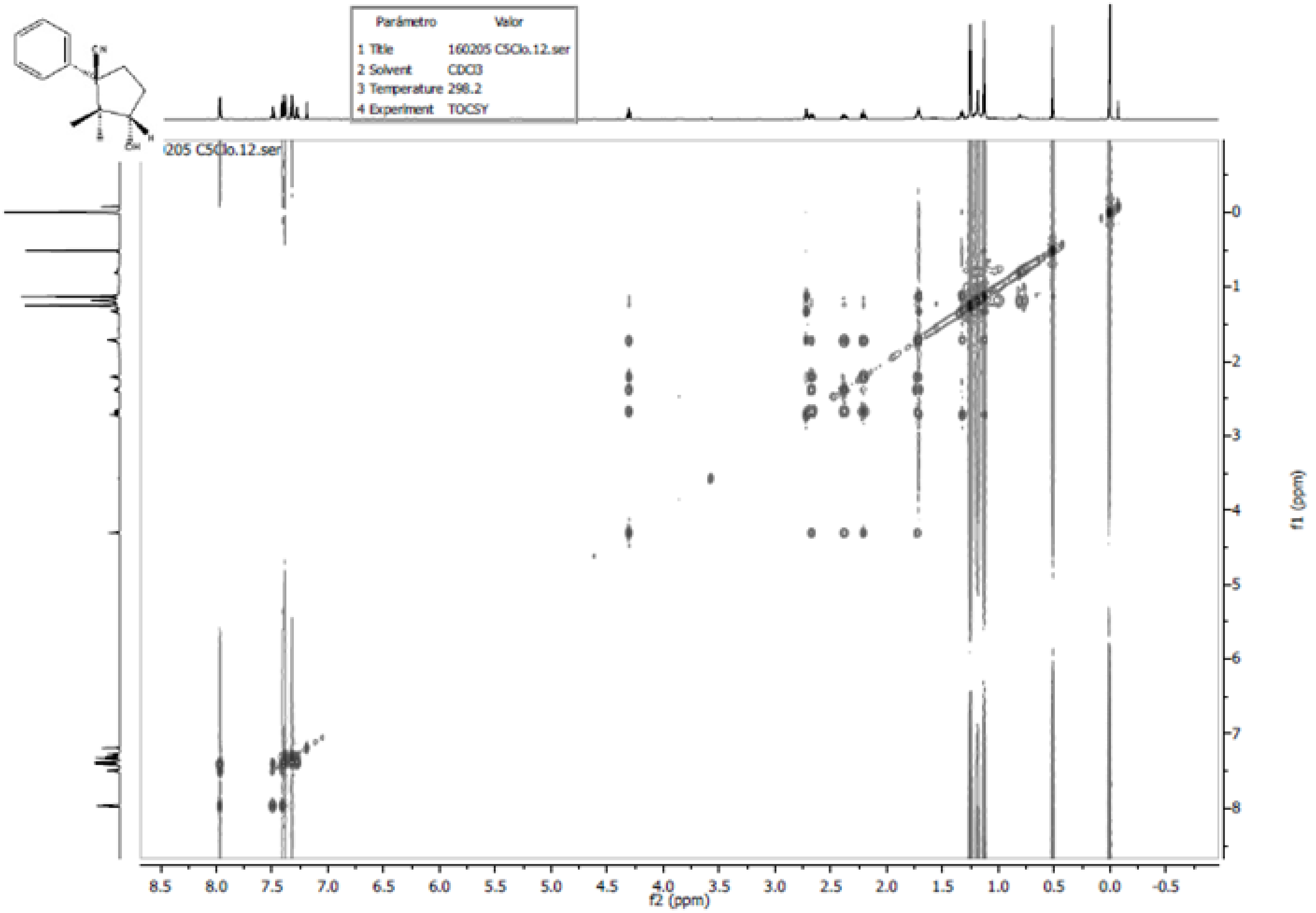


**Figure S25.** TOCSY 3-hydroxy-2,2-dimethyl-1-phenylcyclopentane-1-carbonitrile

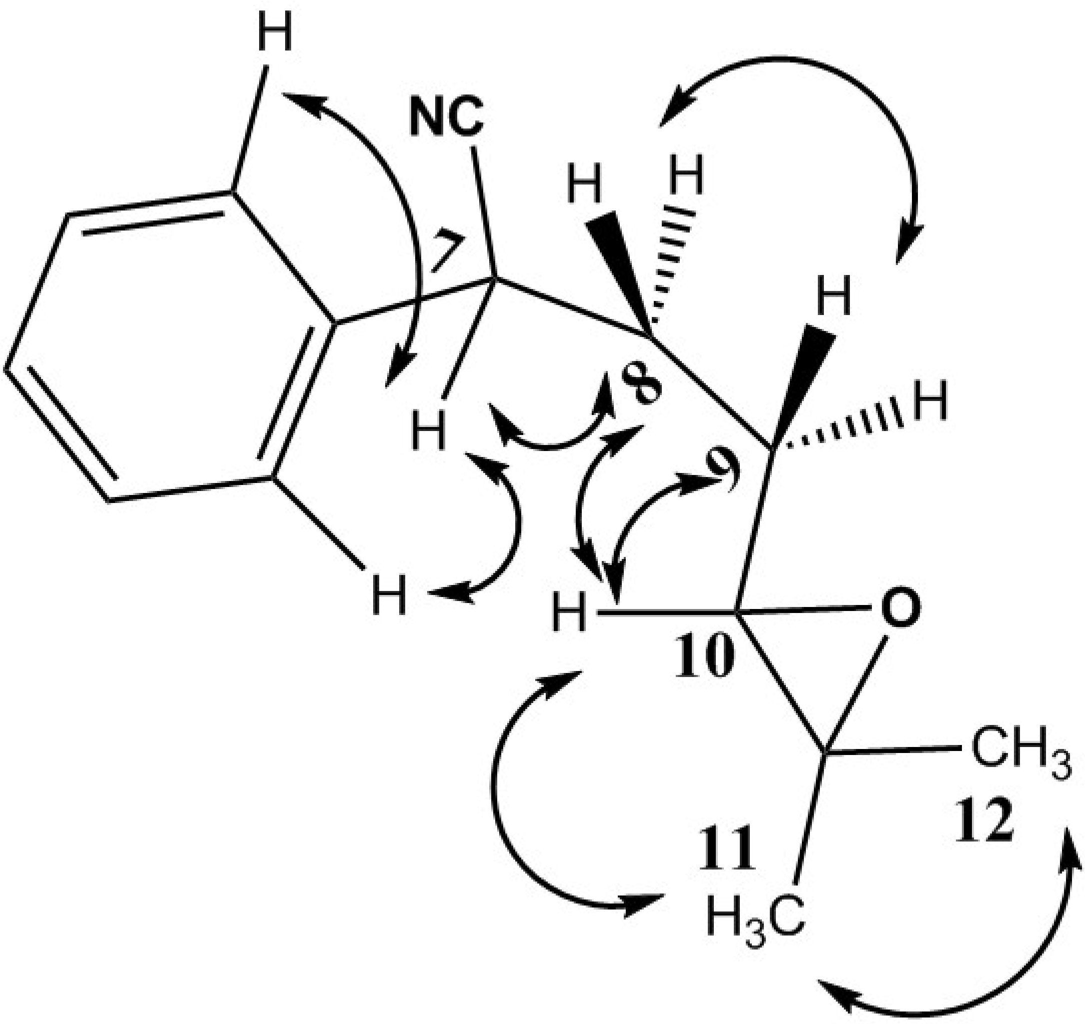


**Figure S26.** Key NOE correlation 4-(3,3-dimethyloxiran-2-yl)-2-phenylbutanenitrile

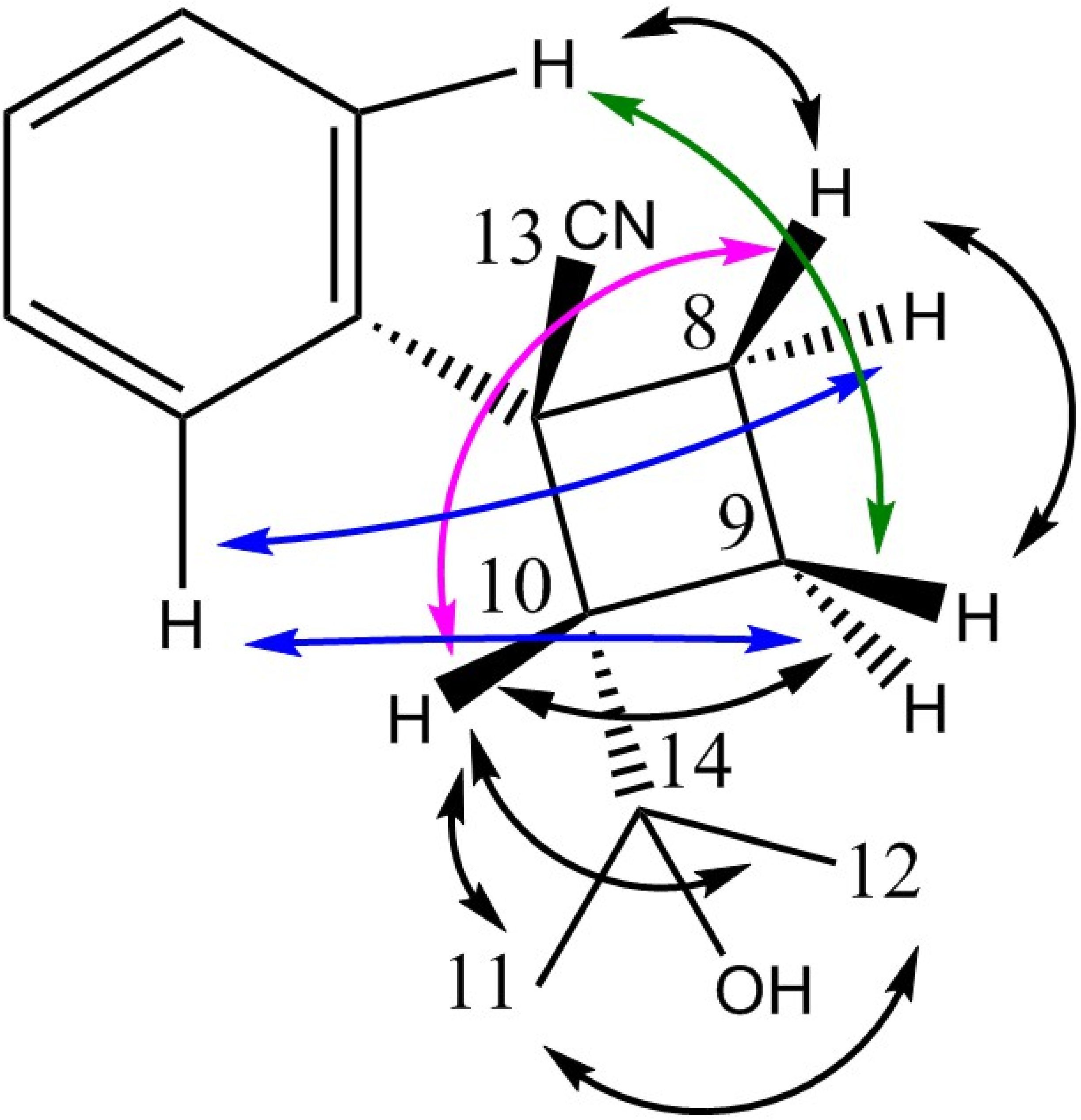


**Figure S27.** Key NOE correlation 2-(2-hydroxypropan-2-yl) 1-phenylcyclobutane-1- carbonitrile

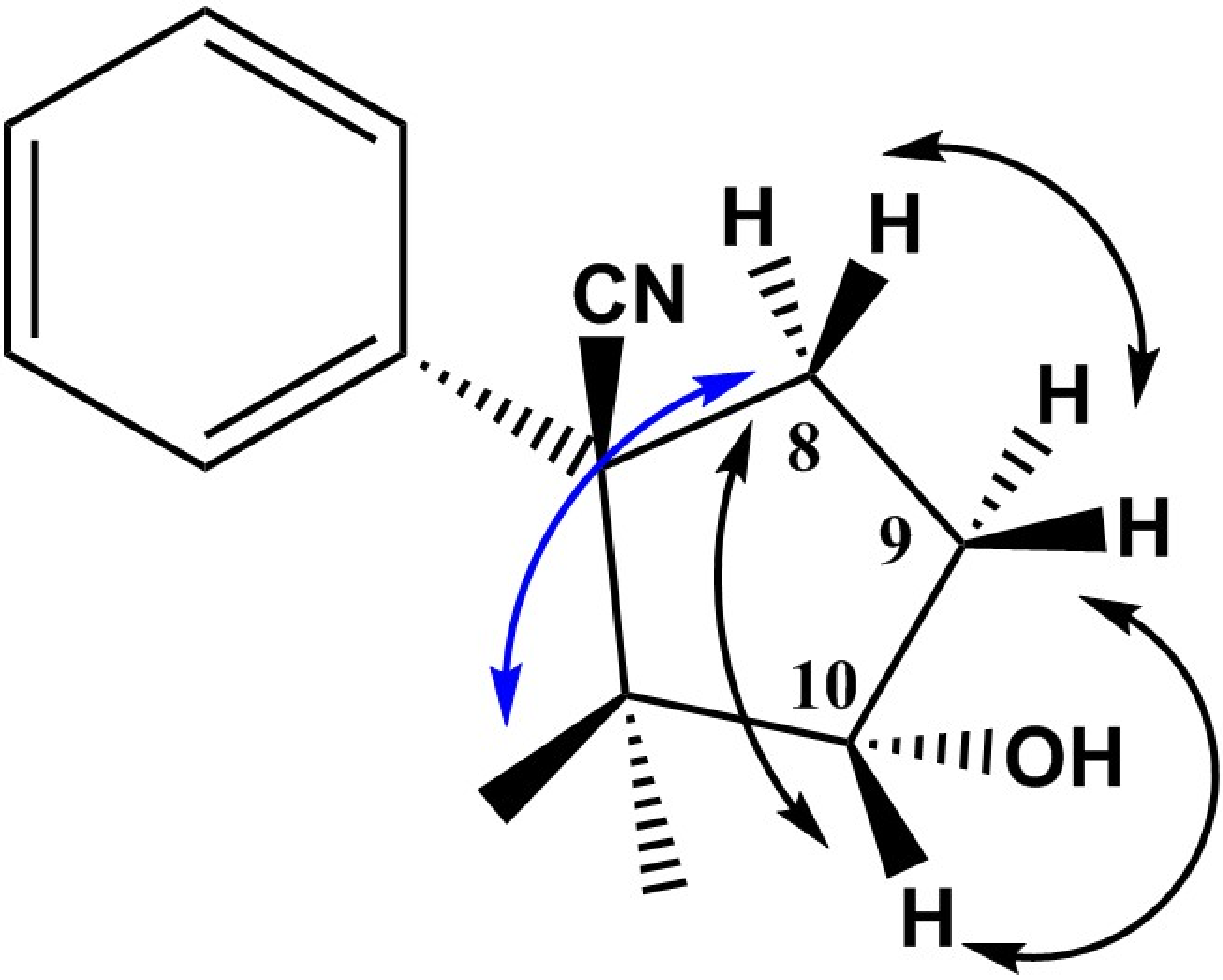


**Figure S28.** Key NOE correlation 3-hydroxy-2,2-dimethyl-1-phenylcyclopentane-1-carbonitrile

## 1.5 Anisotropic titration

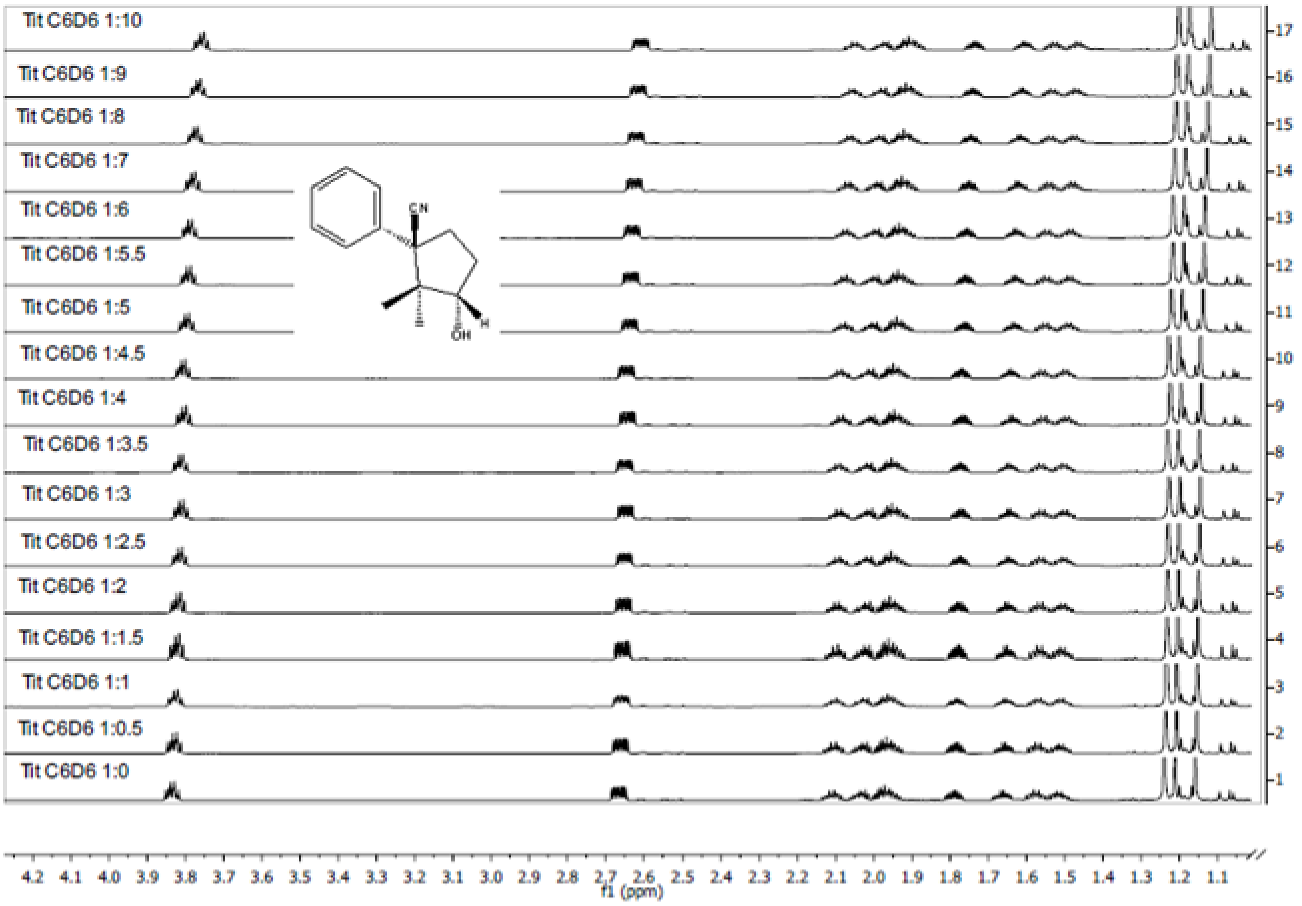


**Figure S29.** 4-(3,3-dimethyloxiran-2-yl)-2-phenylbutanenitrile ($CDCl_3$, benzene-$d^6$ additions)

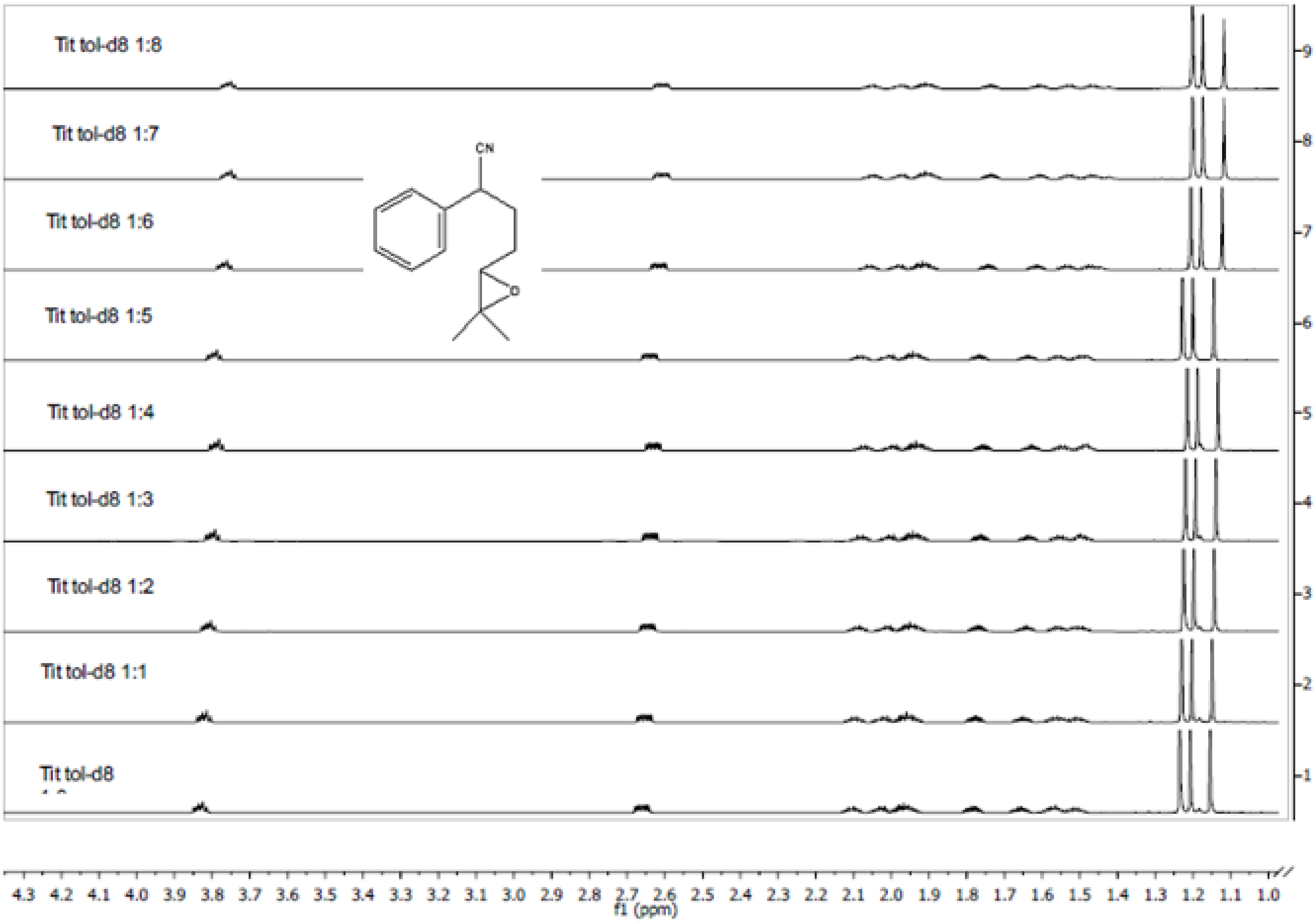


**Figure S30.** 4-(3,3-dimethyloxiran-2-yl)-2-phenylbutanenitrile ($CDCl_3$, toluene-$d^8$ additions)

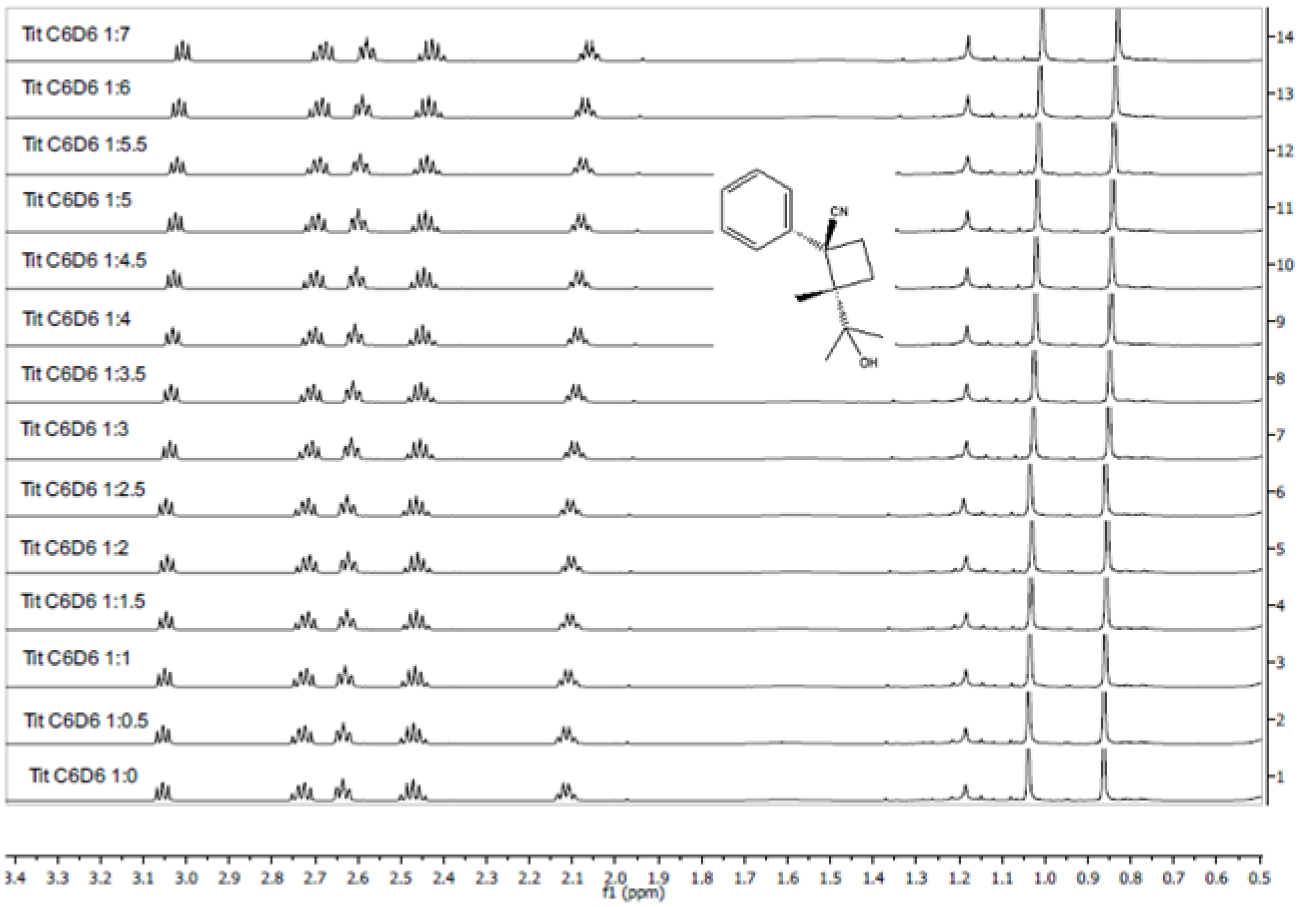


**Figure S31.** 2-(2-hydroxypropan-2-yl) 1-phenylcyclobutane-1- carbonitrile ($CDCl_3$, benzene-$d^6$ additions)

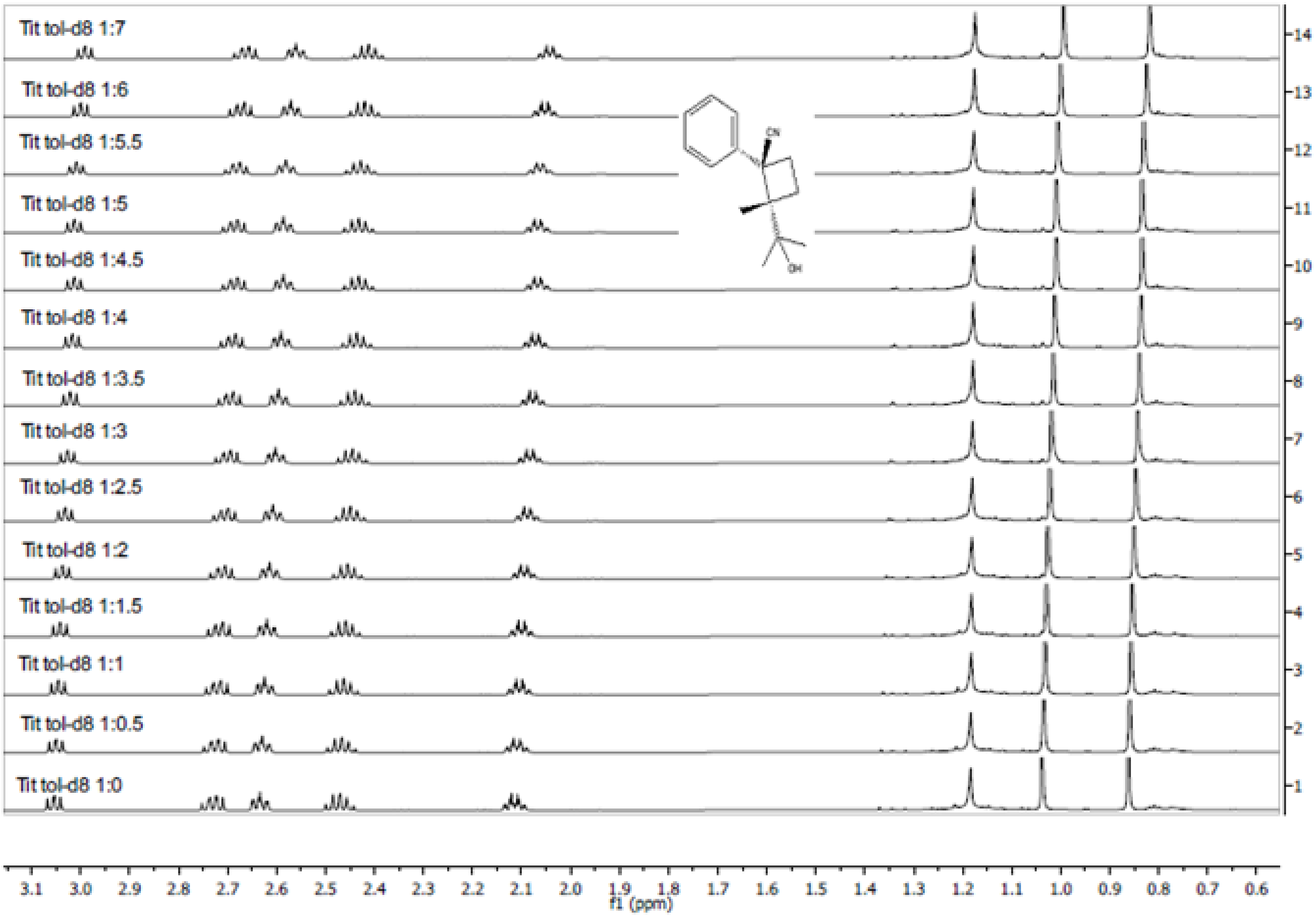


**Figure S32.** 2-(2-hydroxypropan-2-yl) 1-phenylcyclobutane-1- carbonitrile ($CDCl_3$, toluene-$d^8$ additions)

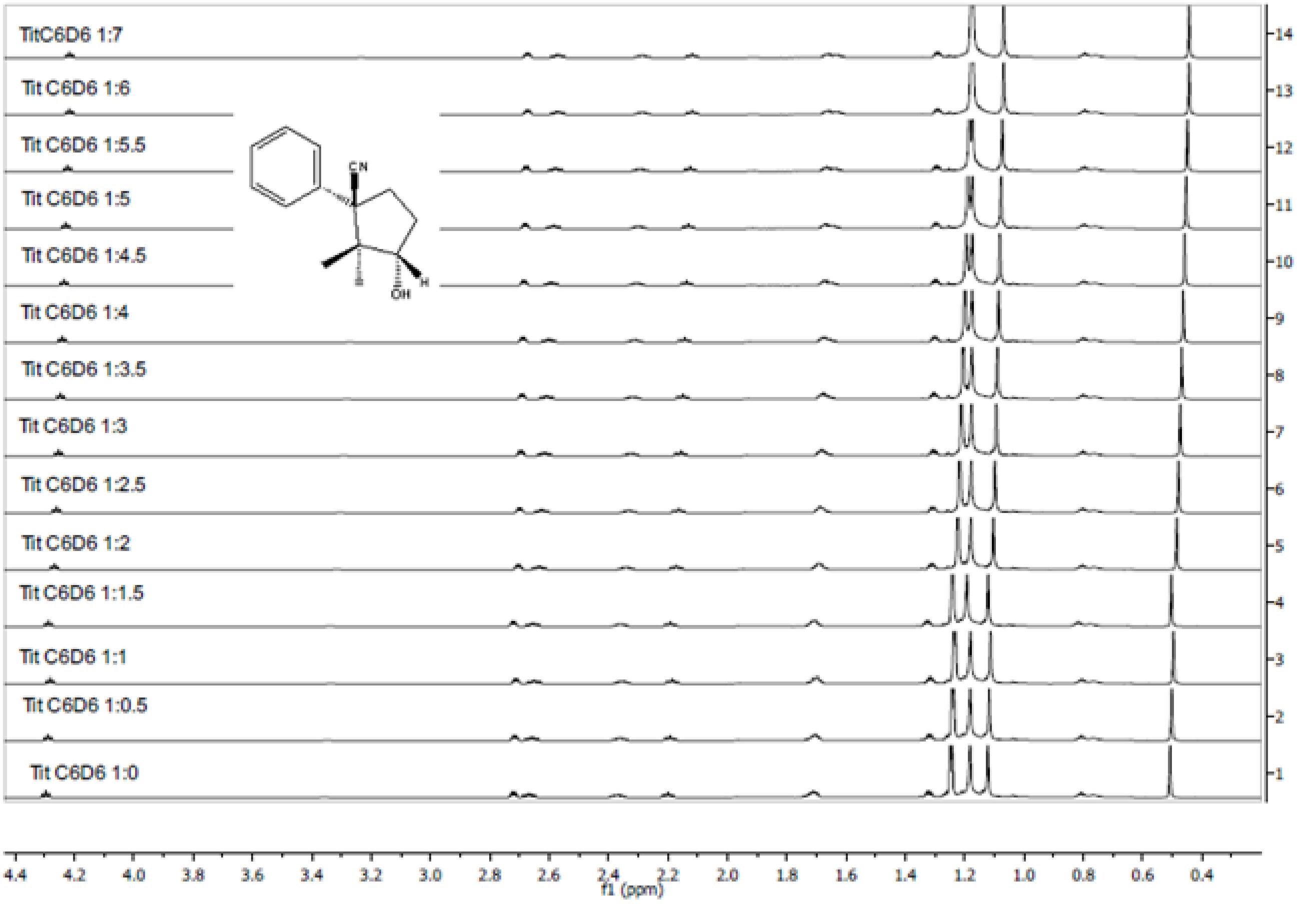


**Figure S33.** 3-hydroxy-2,2-dimethyl-1-phenylcyclopentane-1-carbonitrile ($CDCl_3$, benzene-$d^6$ additions)

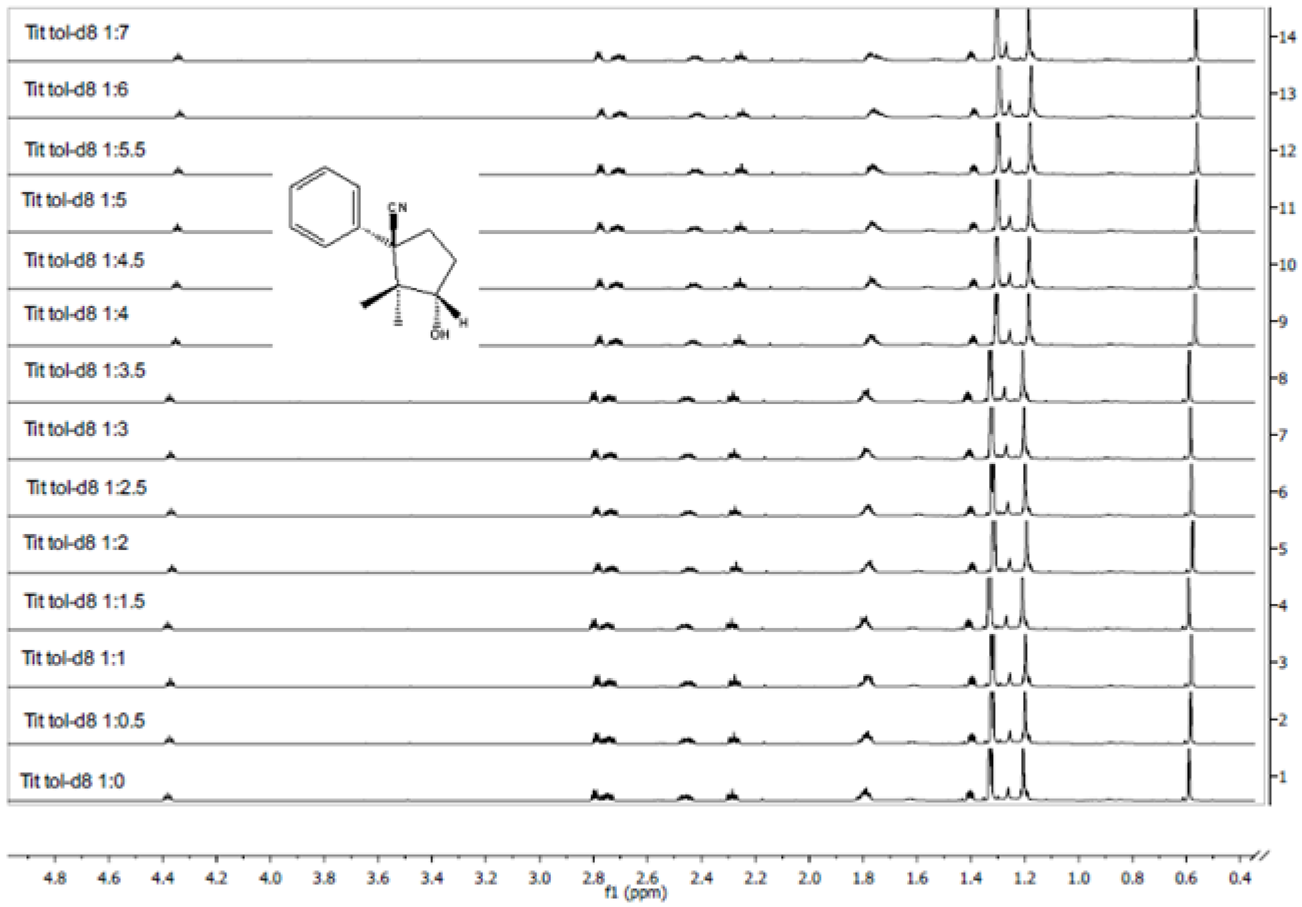


**Figure S34.** 3-hydroxy-2,2-dimethyl-1-phenylcyclopentane-1-carbonitrile ($CDCl_3$, toluene-$d^8$ additions)

## 1.6 Calorimetric determinations

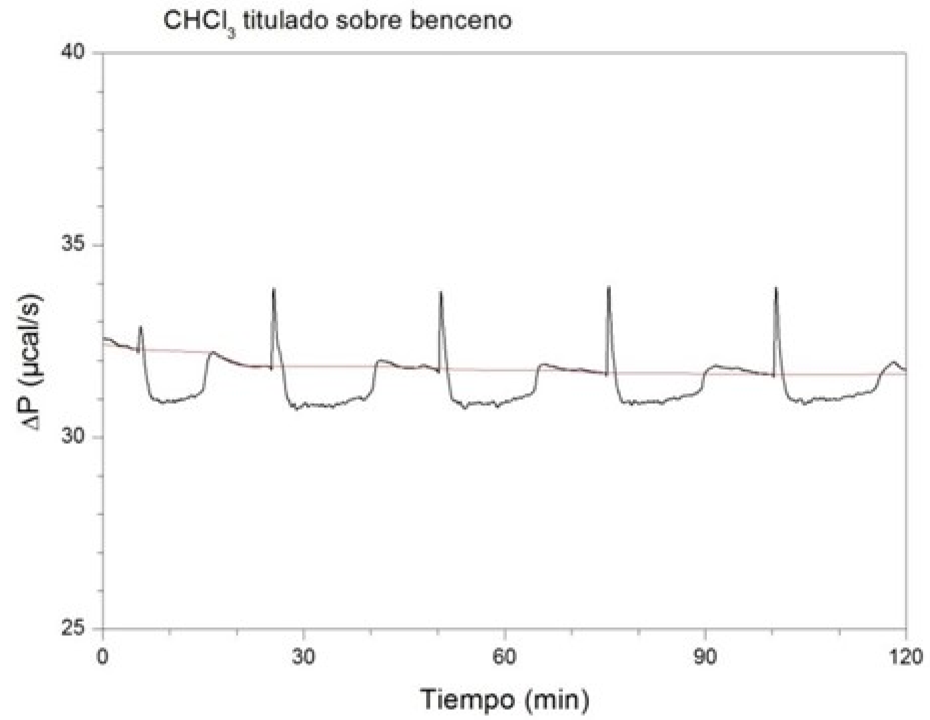


**Figure S35.** CHCl3 determination in benzene.

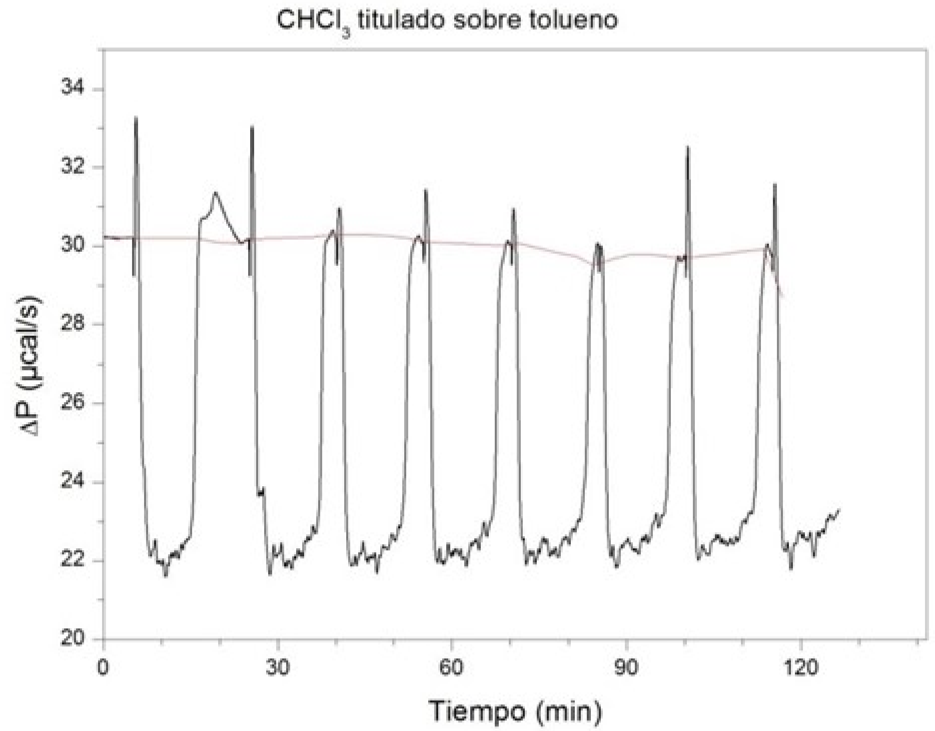


**Figure S36.** CHCl3 determination in toluene.

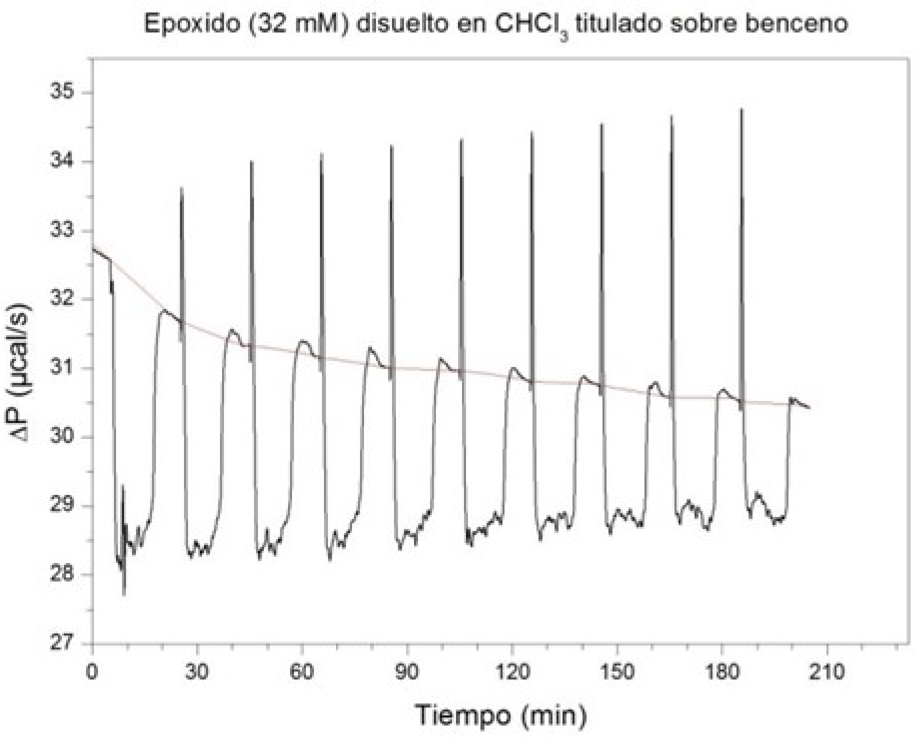


**Figure S37.** Compound 1 solvated in CHCl3, benzene additions

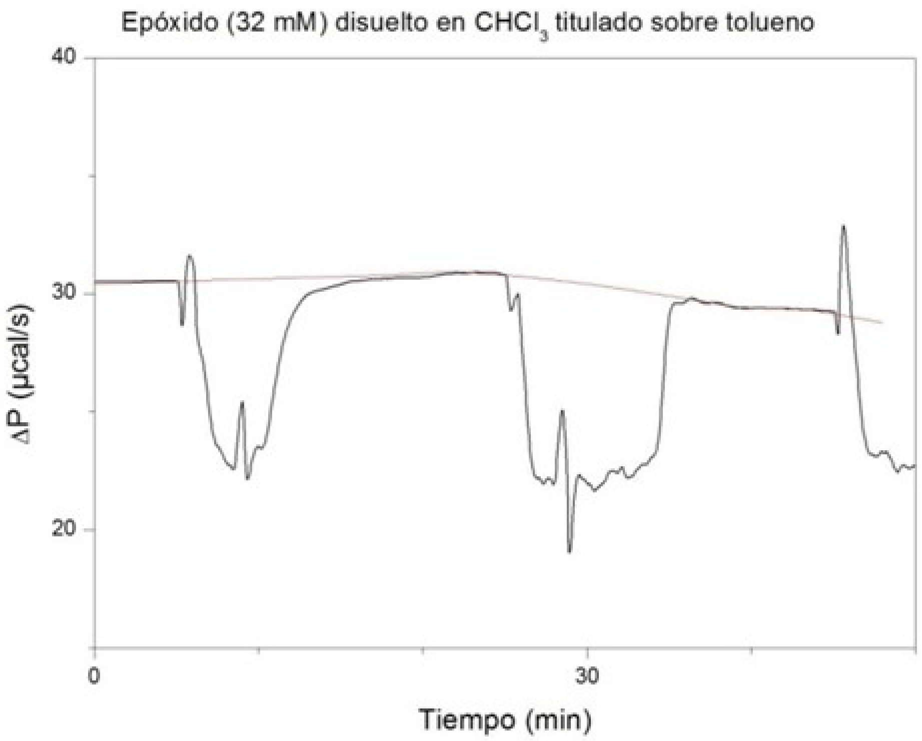


**Figure S38.** Compound 1 solvated in CHCl3, toluene additions.

## 1.7 IRC

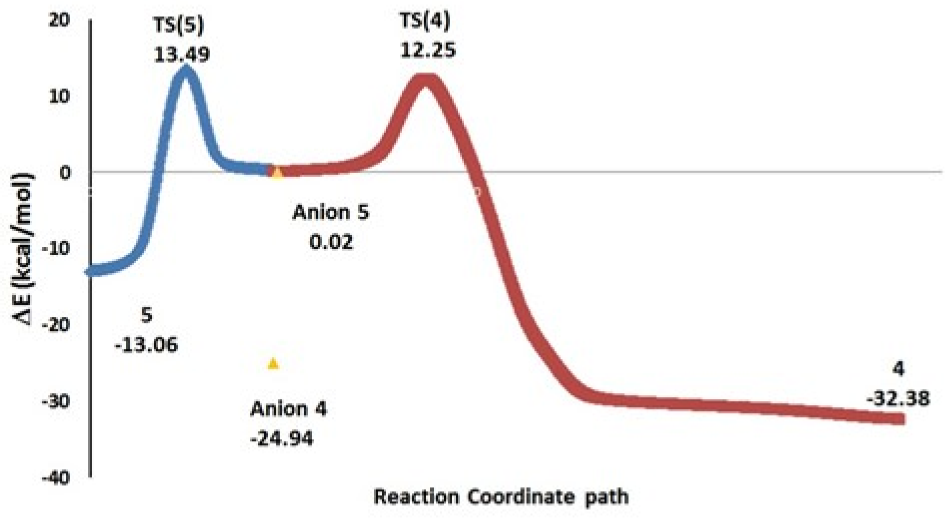


**Figure S39.** IRC in gas phase for both reaction paths.

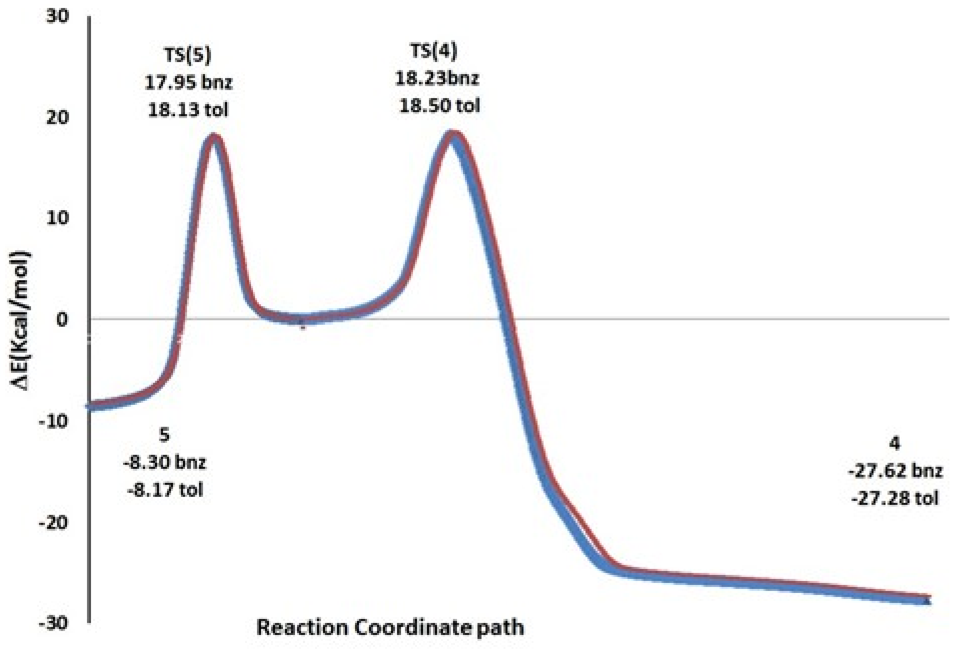


**Figure S40.** IRC in continuum solvent for both reaction paths.

## REFERENCES

Fieser, M. (1967). *Reagents for organic synthesis*, vol. 1 (John Wiley & Sons)

Julia, M. and Ward, P. (1973). Synthesis using sulfones. 4. synthesis of alpha-santalene and alpha-santalol. *BULLETIN DE LA SOCIETE CHIMIQUE DE FRANCE PARTIE II-CHIMIE MOLECULAIRE ORGANIQUE ET BIOLOGIQUE* , 3065–3067

Kofron, W. G. and Hauser, C. R. (1970). Coupling of carbanions. formation of succinic acid derivatives. *J. Org. Chem.* 35, 2085–2086

Lujan-Montelongo, J. A., Vazquez-Sanchez, A., and Avila-Zarraga, J. G. (2009). Regio and stereo selectivity on the $\alpha$-aryl-$\delta$-epoxynitrile anionic cyclization reactions: modulation towards a 5-endo or 4-exo process. *Heterocycles* 78, 1955–1976
